# Polyvalent guide RNAs for CRISPR antivirals

**DOI:** 10.1016/j.isci.2022.105333

**Published:** 2022-10-13

**Authors:** Rammyani Bagchi, Rachel Tinker-Kulberg, Mohammad Salehin, Tinku Supakar, Sydney Chamberlain, Ayalew Ligaba-Osena, Eric A. Josephs

**Affiliations:** 1 Department of Nanoscience, The University of North Carolina at Greensboro, Greensboro, NC 27401, USA; 2 Department of Biology, The University of North Carolina at Greensboro, Greensboro, NC 27401, USA

**Keywords:** Biocomputational method, Computational bioinformatics, Biological sciences tools

## Abstract

CRISPR effector Cas13 recognizes and degrades RNA molecules that are complementary to its guide RNA (gRNA) and possesses potential as an antiviral biotechnology because it can degrade viral mRNA and RNA genomes. Because multiplexed targeting is a critical strategy to improve viral suppression, we developed a strategy to design of gRNAs where individual gRNAs have maximized activity at multiple viral targets, simultaneously, by exploiting the molecular biophysics of promiscuous target recognition by Cas13. These “polyvalent” gRNA sequences (“pgRNAs”) provide superior antiviral elimination across tissue/organ scales in a higher organism (*Nicotiana benthamiana*) compared to conventionally-designed gRNAs—reducing detectable viral RNA by >30-fold, despite lacking perfect complementarity with either of their targets and, when multiplexed, reducing viral RNA by >99.5%. Pairs of pgRNA-targetable sequences are abundant in the genomes of RNA viruses, and this work highlights the need for specific approaches to the challenges of targeting viruses in eukaryotes using CRISPR.

## Highlights

• Algorithm to design single gRNAs to target multiple viral sequences simultaneously
• Pairs of targetable sequences are abundant in the genomes of RNA viruses
• Validated in plants with Cas13, reducing viral RNA >30-fold versus conventional gRNAs
• Approach also works in viral diagnostics and with other CRISPR effectors like Cas9

## Graphical abstract

**Figure:**
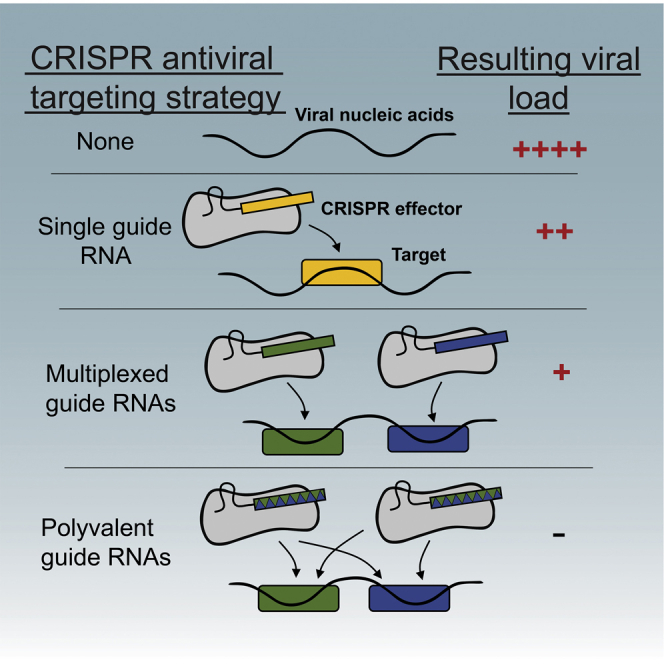

## Introduction

**Figure 1:**
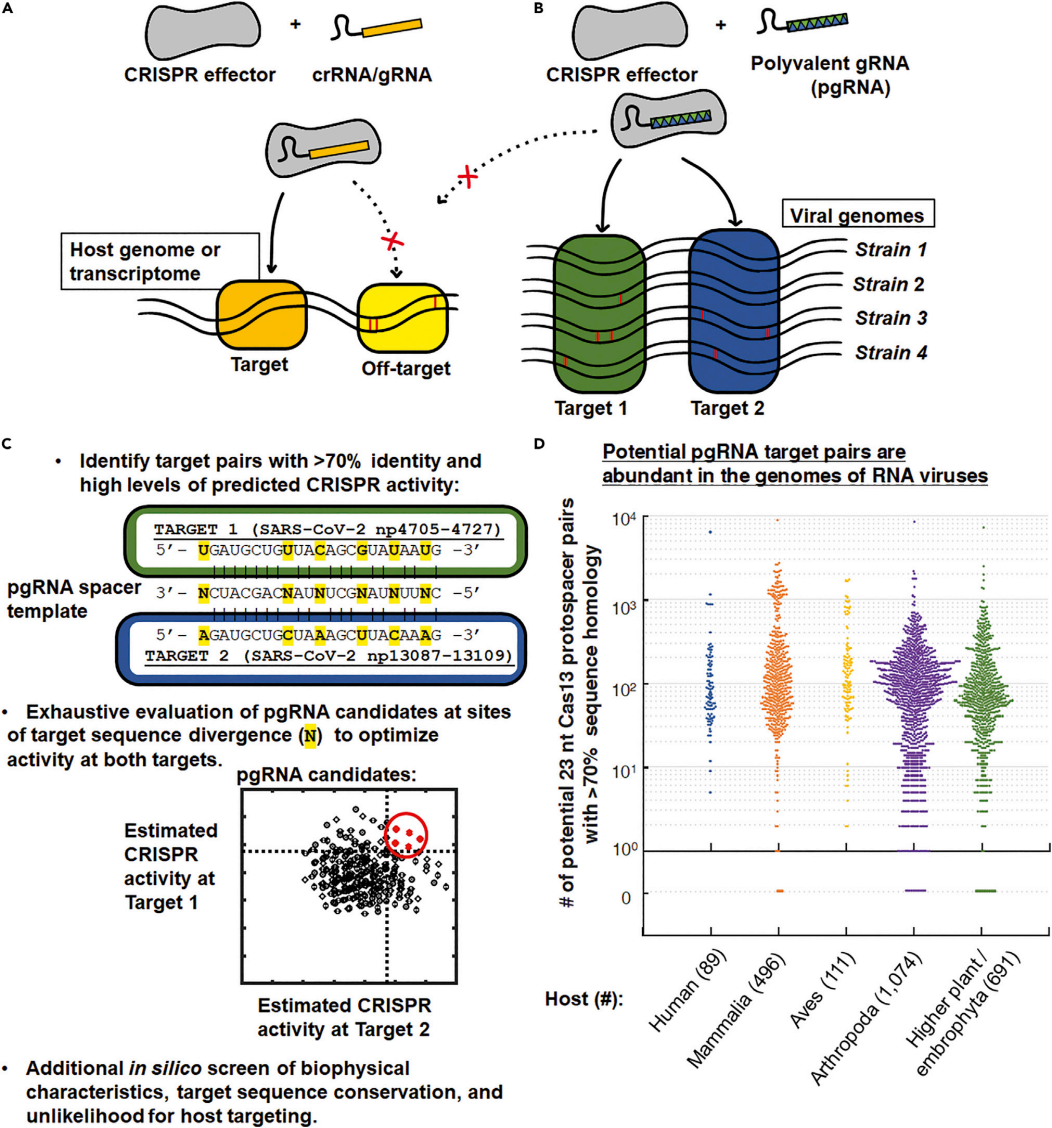
“Polyvalent” guide RNAs for CRISPR antiviral biotechnologies (A and B) Precision gene editing applications of CRISPR require extreme specificity in mutating only a single target site; however, (B) for antiviral applications of CRISPR, recognition and degradation of multiple viral targets is advantageous for suppressing viral propagation, expanding recognition of strain variants, improving viral detection sensitivity, and preventing mutagenic escape. A single “polyvalent” guide RNA (pgRNA) may lack perfect complementarity with their targets but are optimized to promote activity at multiple viral sites, simultaneously, while avoiding the host genome or transcriptome. (C) Protocol for designing pgRNAs: After target pairs with >70% homology have been identified in the same viral genome, the nucleotides at positions where the sequence between the two targets differ are chosen to minimize potential reductions of activity at the different sites by determining which mismatch- and position-specific mispairings are best-tolerated by the CRISPR effector. See Figure S1 and STAR methods for further details. (D) Pairs of targetable sites for Cas13 (23 nt), which share at least 70% homology, are abundant across the genomes of RNA viruses. All complete, RefSeq-quality genomes of RNA viruses, excluding proviruses, available by December 27, 2020 were downloaded from the NCBI Virus database with hosts (each point): arthropoda (1074 viral species), aves (111), mammal (496), higher plant/embrophyta (691), and human (89). Genomes composed of multiple segments or CDS from the same viral isolate were considered together. See also Figure S2A.

Class II CRISPR effectors (Figure 1) like Cas9, Cas12, and Cas13, are nucleases that use a modular segment of their RNA cofactors known as CRISPR RNAs (crRNAs) or guide RNAs (gRNAs) to recognize and trigger the degradation of nucleic acids with a sequence complementary to that segment (Shmakov et al., 2017). Because of their ability to be easily redirected to nucleic acids with different sequences by simply changing the sequence composition of a short portion of their gRNAs called their ‘spacer,’ CRISPR-based biotechnologies have been rapidly developed over the past several years for a number of different applications, most notably in precision gene editing (Figure 1A) (Knott and Doudna, 2018).

Beyond gene editing, another nascent but less developed application of CRISPR effectors has been as novel antiviral diagnostics, prophylactics, and therapeutics, based on their ability to recognize and degrade viral nucleic acids and genetic material (Soppe and Lebbink, 2017). Because the vast majority of pathogenic viruses are RNA viruses, RNA-guided RNA endonucleases like type VI CRISPR effectors Cas13a (formerly C2c2), Cas13b, and Cas13d hold significant promise for CRISPR antiviral biotechnologies (Freije et al., 2019; Gootenberg et al., 2017; Myhrvold et al., 2018; Yan et al., 2018). Through their targeted degradation of viral single-stranded RNA (ssRNA) genomes or mRNA, Cas13 variants have been recently shown to reduce viral burdens in plants and plant cells (Abudayyeh et al., 2016; Aman et al., 2018; Mahas et al., 2019), mammalian cells (Li et al., 2020a, 2020b), and human (Abbott et al., 2020; Freije et al., 2019) cells, as well as more recently in animal models (Blanchard et al., 2021). Type II CRISPR effector Cas9 and type V CRISPR effector Cas12, which recognize and introduce breaks into double-stranded DNA (dsDNA) targets, have also been used *in vitro* (Gao et al., 2020; Lebbink et al., 2017; Wang et al., 2018) and more recently in animal models (Yin et al., 2021) to degrade dsDNA viruses and to excise proviruses from cells with latent retroviral infection (Lee, 2019). In addition, both type V and type VI CRISPR effectors exhibit nonspecific ssDNAse and ssRNAse activity after recognition of their targets, respectively, and this nonspecific activity has been harnessed for viral detection in diagnostic devices (Broughton et al., 2020; Freije et al., 2019; Gootenberg et al., 2018; Myhrvold et al., 2018). Although all of these applications have shown significant promise for the future of CRISPR antivirals, further maturation of these biotechnologies is required before they can reach their full potential.

For example, there are several major challenges to the development of effective CRISPR-based antiviral biotechnologies—challenges which are different from those that arise during the development of effective gene editing biotechnologies for Cas13-based knockdown of mRNA expressed from eukaryotic cells (Konermann et al., 2018)—that result from the rapid proliferation and mutation rates of viruses. For example, compared even to mRNA knock-down experiments in human cells, RNA viruses are a “moving target”: they rapidly replicate inside of a cell; many copies of the viral genome are used to generate very high levels of viral mRNA (vmRNA) in order to generate significant amounts of viral proteins (Lindbo, 2007); and viral RNA genomes rapidly mutate while they replicate—thought to be around one mutation per viral replication. While maintaining very high levels of targeted nuclease activity and while still avoiding any potential host nucleic acids, CRISPR-based antiviral biotechnologies must therefore also be able to robustly target a specific virus where sequence polymorphisms may exist across strains or clinical variants and, relatedly, must also be able to suppress mutational escape, where the emergence of novel mutations can limit or eliminate the ability of the CRISPR effector to recognize the viral genome. These challenges have primarily been addressed using two approaches: by targeting the CRISPR effector to regions of high sequence conservation in the viral genome (Abbott et al., 2020; Blanchard et al., 2021; Darcis et al., 2019; Freije et al., 2019; Guo et al., 2021), and by introducing multiple gRNAs to target different segments of the viral genome simultaneously (multiplexing) to make viral escape less likely (Abbott et al., 2020; Freije et al., 2019; Guo et al., 2021; Lebbink et al., 2017). Overall, the design of effective gRNAs for antiviral applications still remains a subject of continued research, but it is clear that their design criteria are differentiated from other applications of CRISPR biotechnologies, and gRNA designs that are best for other applications of CRISPR biotechnologies might not be optimal for antiviral applications.

We propose an alternative strategy for the design of gRNAs for CRISPR antivirals that exploits the widely-recognized tendency of different CRISPR effectors to possess varying levels of tolerance to imperfect complementary between the gRNA spacer and the targets (Doench et al., 2016; Fareh et al., 2021; Listgarten et al., 2018; Wessels et al., 2020) in order to maximize their antiviral potential. Given that multiplexed targeting is a critical tactic for inhibiting viral infection (Yin et al., 2021), expanding the recognition of clinical strain variants (Darcis et al., 2019; Guo et al., 2021), improving viral detection sensitivity (Fozouni et al., 2021), and limiting mutagenic escape from CRISPR antivirals (Lebbink et al., 2017), we hypothesized that if we could match target sequences in a viral genome to other targets with some shared sequence homology in the same viral genome, a single gRNA spacer sequence could be optimized for CRISPR ribonucleoprotein (RNP) activity at more than one specific target (Figure 1B). We hypothesized that at sets of viral targets that shared some homology but were not necessarily identical, the spacer sequence of a gRNA could be altered in such a way that, despite not having perfect complementary to any of targets, the CRISPR RNPs would still be able to recognize and degrade all members of that target set. We also hypothesized that this “polyvalent” activity or activity at multiple sites by a single gRNA would not just compensate for any potential reductions in activity at the target sites that result from any mispairings between the spacer and the targets, but rather would enhance their antiviral potential by increasing the effective number of “targets” on a virus that can be recognized per effector—which could help to increase the rate of viral recognition by each CRISPR RNP, improve the probability of generating a disabling mutation and/or limit viral mutagenic escape as if multiplexed targeting was being performed, and reduce the components required for this operative multiplexed targeting. Lastly, because of the number of possible spacer sequences is huge, we hypothesized that spacer sequences that could result in “polyvalent” activity at multiple viral sequences could still be designed while still maintaining extreme levels of divergence from host sequences in order to limit any potential interactions with the host genome or transcriptome during a potential viral treatment (Figure 1B). Altogether, we reasoned that, despite imperfect complementarity to the virus, “polyvalent” gRNAs (pgRNAs) may be better for CRISPR antiviral biotechnologies than perfectly-complementary “monovalent” gRNAs and developed a computational algorithm to design spacer sequences with these properties (promoting strong activity at multiple viral targets while maintaining high levels of sequence divergence with any host genomic or transcriptomic sequences) (Figures 1C and S1).

The process of engineering gRNAs with “polyvalent” activity is, in some respects, the opposite as what is performed during gRNA design for applications in precision gene editing and transcriptome engineering, where significant efforts have gone toward limiting the tendency for CRISPR RNP activity at sequences with imperfect complementarity and particularly toward preventing mutagenic CRISPR activity at multiple or unintended “off-target” sites at all costs (Figure 1A). However, gRNAs that are predicted to elicit significant activity by the CRISPR RNP at multiple sites would normally be algorithmically rejected by gRNA design tools that were created primarily for use in gene editing (Listgarten et al., 2018), so new approaches that were optimized for antiviral applications were necessary.

Here, we present a computational algorithm to efficiently design pgRNA sequences optimized for activity at multiple viral targets, simultaneously, and experimentally demonstrate that using pgRNAs with Cas13 in a higher organism (*Nicotiana benthamiana,* model tobacco) can provide superior antiviral elimination versus an infectious RNA virus compared to conventionally-designed gRNAs. We show that (1) despite lacking perfect complementarity to their targets, single pgRNAs with Cas13 targeting two viral sites can robustly suppress viral propagation in whole *N. benthamiana* plants across the organ/tissue scale, (2) that treatments with pgRNAs are better than those using a single perfectly-complementary “monovalent” gRNA—in some cases further reducing detectable viral RNA by an additional order of magnitude (30- to 40-fold) compared with “monovalent” gRNAs—and with fewer components single pgRNAs are at least as effective as and often better than treatments using two of their multiplexed “monovalent” gRNA counterparts; and (3) that treatments using multiplexed pgRNAs (using two gRNAs to target four viral sequences) perform even better, reducing detectable viral RNA *in planta* by >99.5%. *in vitro*, we show that, despite imperfect complementarity with their targets, pgRNAs can be engineered to trigger Cas13’s “collateral activity” for viral detection applications at viral target pairs with sequences diverging by up to 25% and that they can be designed to direct DNA-targeting CRISPR-Cas9 to degrade multiple DNA targets *ex vivo* with sequence divergence up to 40% (with mismatches at 8 out of 20 bp).

We emphasize that pgRNAs are engineered to target two specific viral sites, not to generally allow their CRISPR effector to be more prone to “off-target activity” or to tolerate more mismatches than any other gRNA or CRISPR effector using conventional gRNA designs: both Cas13 and Cas9 have well-characterized natural tolerances to mismatches between their gRNA spacer sequence and targets that we exploit for the specific targeting of two selected viral sites. Pairs of sequences that are simultaneously CRISPR-targetable by pgRNAs are abundant in viral genomes (Figures 1D and S2A) and, overall, the results presented here represent a broadly-applicable and powerful paradigm for “polyvalent” gRNA design in antiviral applications that specifically addresses some of the differential requirements for CRISPR antivirals compared to those for precision gene editing.

## Results

### Design of “polyvalent” guide RNAs (pgRNAs) for CRISPR antiviral biotechnologies

The algorithm to design pgRNAs (Figures 1C and S1, and STAR methods) starts by identifying pairs of protospacers (the nucleotide targets of a CRISPR RNP) in the genome of a virus of interest where the target pairs shared at least 70% sequence homology and where levels of CRISPR RNP activity at both targets were computationally predicted to be within the top quartile (Doench et al., 2014; Wessels et al., 2020) for all predicted protospacers targeting the viral genome. An analysis of 2,372 genomes of RNA viruses in the NCBI Reference Sequence database (Hatcher et al., 2017) revealed that these homeologous pairs of Cas13-targetable sites (23 nt) with >70% identity (>16 out of 23 nt) are prevalent across RNA viruses of mammals, birds, arthropods, and plants (Figures 1D and S2A): RNA viruses with genomes that are 5,000 nt in length have on average around 30 of such pairs, and those with genomes that are 10,000 nt in length have on average approximately 120, obeying a power law scaling with genome length (Figure S2A and Table S1: Statistics of Homeologous Cas13 Target Pair (>16/23 or 70% sequence identity) Prevalence in RNA viral genomes). For human-hosted RNA viruses, we could identify 19,926 of these homeologous target pairs across 89 viruses (Table S2: Homeologous Cas13 Target Pairs (>16/23 or 70% sequence identity) of Human-hosted RNA viruses).

**Figure 3:**
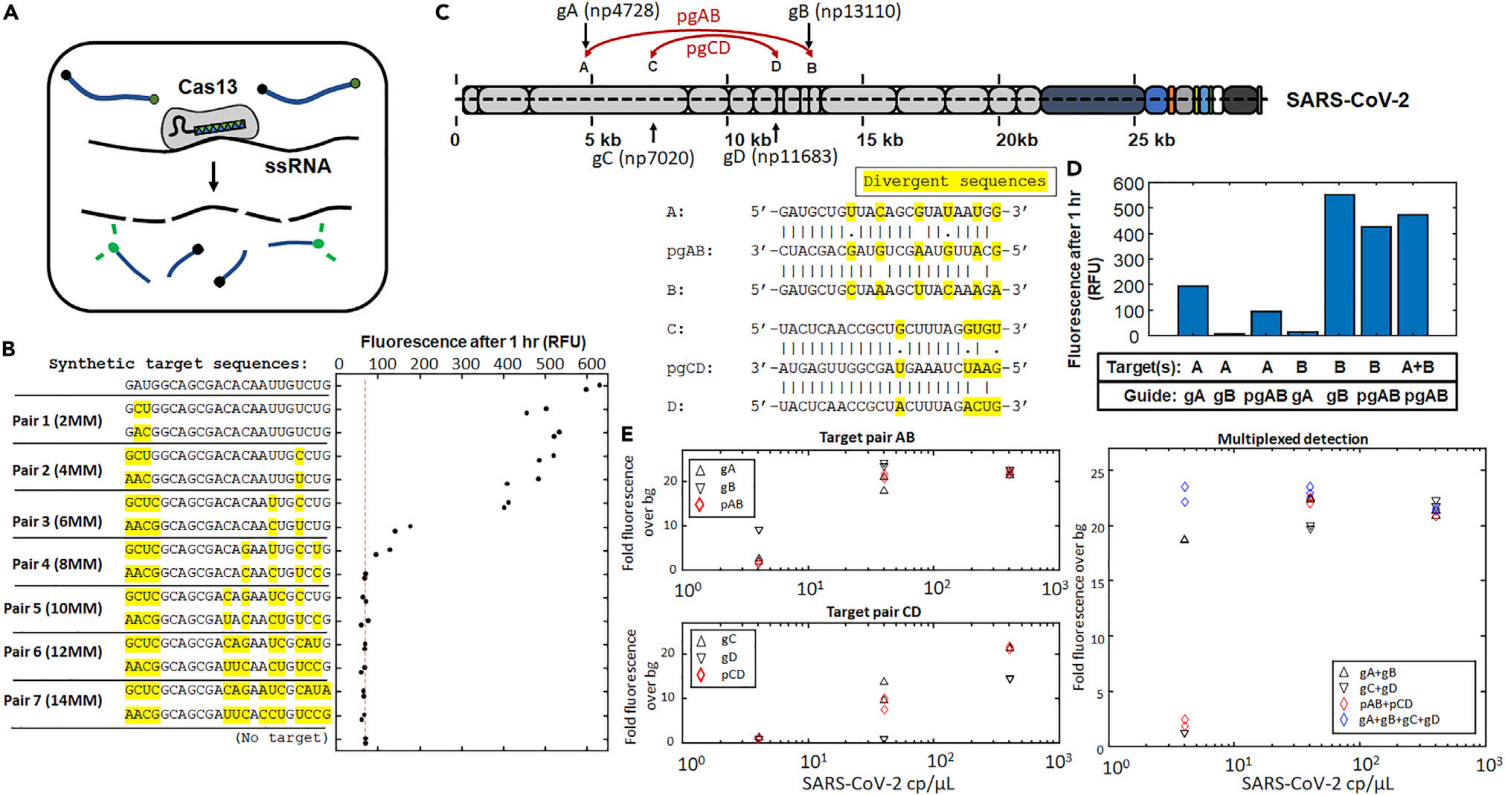
pgRNAs can be engineered to stimulate “collateral activity” by Cas13 for viral detection *in vitro* (A) After recognizing a target, Cas13 exhibits nonspecific RNAse activity; nonspecific degradation of a fluorescent reporter RNA results in a fluorescent signal that can be detected in viral diagnostic assays. (B) Detectable collateral activity is stimulated by Cas13 *in vitro* at targets with sequence divergence up to 25%. (C) pgRNAs were designed to target (+) ssRNA virus SARS-CoV-2, see Tables S10 and S11 for a screen of 23 pgRNAs at 15 target-pairs for the highest collateral activity in the presence of both sites (pgAB and pgCD). The sequences of the pgRNAs are optimized for activity at both targets, even with predicted mispairings and non-canonical base pairings with each target. (D) Monovalent gRNAs exhibit no cross-reactive collateral activity, while pgRNAs exhibit collateral activity in the presence of either SARS-CoV-2 target. (E) In an SHERLOCK-type Cas13 viral diagnostic assay, Cas13 with single pgRNAs (recognizing two sites, left) or two pgRNAs (recognizing four, right) could robustly generate detectable signals in the presence of samples initially containing 40 cp/uL heat-inactivated SARS-CoV-2 (clinically relevant LoD for SARS-CoV-2 is often considered to be 1000 cp/uL).

Candidate pgRNA sequences for each pair are then generated *in silico* by determining what nucleotides at the positions of divergent sequence between the two targets would allow for and maximize predicted activity at both sites (Figure 1C), which is performed by calculating the expected “mismatch penalties” or reduction of CRISPR RNP activity for those candidates at sites with imperfect complementarity to the spacer sequence. Mismatch penalties have been quantitatively determined for several CRISPR effectors (Doench et al., 2016; Listgarten et al., 2018; Tycko et al., 2018; Wessels et al., 2020) and exhibit a strong dependence on both the type of mismatch (what nucleotides are incorrectly paired) and the position of the mismatch(es) along the target: They have been found to vary not only by type of CRISPR effector but across homologues of the effector derived from different species (Figure S3). For the design of pgRNAs, sequences are selected by computationally maximizing the predicted activity of a single gRNA at multiple viral sites by exploiting well-tolerated mismatch- and position-specific mispairings of the CRISPR effectors to minimize potential reductions of activity at the different sites (STAR methods). Sequences with predicted biophysical properties that might negatively impact expression or activity, such as strong predicted secondary structures or the presence of mononucleotide stretches, are then removed from consideration. Furthermore, to prevent “off-target” interactions with the host genome or transcriptome, any sequences with more than 65% complementarity with potential “off-targets” in the host genome or transcriptome (with at least 15 complementary nts in the 23 nt Cas13 spacer) are also rejected, yielding a final set of pgRNA candidates with high predicted activity at multiple viral sites and effectively no predicted “off-target” activity versus the host (Figure 1C). Earlier biochemical studies have shown that Cas13days requires at least 18 nt of complementarity to its target for stable binding and target cleavage to occur (Zhang et al., 2018), and *in vitro*, we have also found (*e.g.,* see Figures 3B–3D) that Cas13 would tolerate no more than 4 mismatches between its gRNA and a ssRNA target before Cas13 RNAse activity is abolished; for pgRNA designs for Cas13 we conservatively require at least 8 mismatches with any host transcripts.

To illustrate the broad potential applicability of our approach, we found we could design pgRNA candidates for RNA-targeting Cas13days from *Ruminococcus flavefaciens* XPD3002 (RfxCas13d) with predicted activity at both their targeted sites ranking in the top quartile of all “monovalent” gRNAs for that virus and no significant homology/predicted activity versus the human transcriptome for 53 of the 59 human-hosted (+) ssRNA viruses or expressed viral mRNA sequences in the NCBI Reference Sequence database (Figure S2B and Table S3: Statistics of pgRNA Candidate Prevalence in human-hosted (+) ssRNA viral genomes and viral RNA transcripts and Table S4: pgRNA Candidate Prevalence in human-hosted (+) ssRNA viral genomes and viral RNA transcripts). RfxCas13d, which has been used in CRISPR-based viral diagnostics (Gootenberg et al., 2018) and was recently demonstrated to disrupt influenza and SARS-CoV-2 virulence in human epithelial cells (Abbott et al., 2020), was found to exhibit significant tolerance to mismatches relative to other CRISPR effectors like Cas9 and Cas12 (Figure S3) and does not require specific flanking sequences next to its targets. DNA-targeting effectors Cas9 and Cas12 also carry an increased risk of introducing permanent genetic mutations into the host genome non-specifically, so RNA-targeting RfxCas13d may represent an optimal and safer effector for therapeutic antiviral applications in that regard whenever possible.

### pgRNAs with RfxCas13d robustly suppress viral spread across the tissue/organ-scales in a higher organism (*N. benthamiana*)

**Figure 2:**
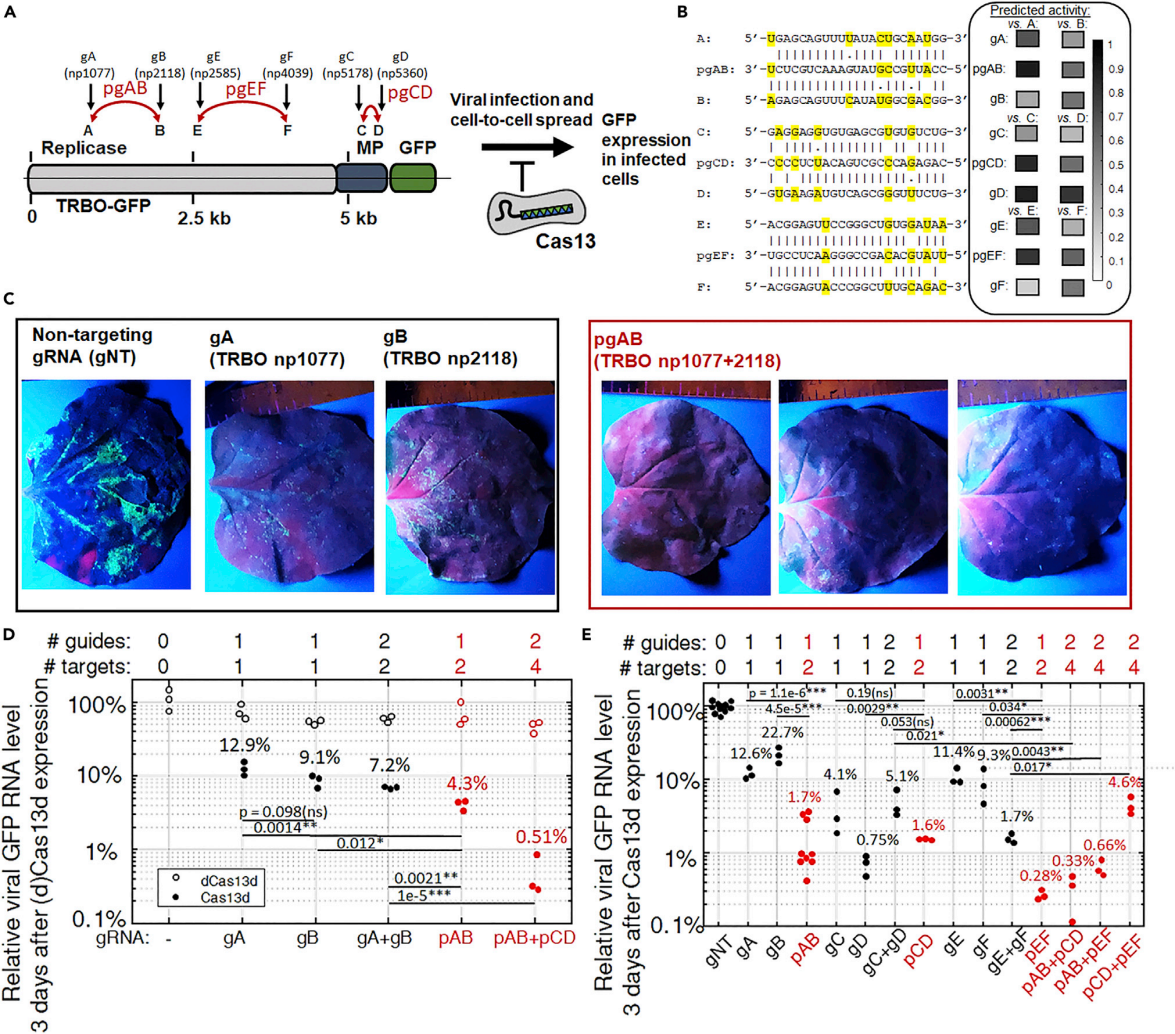
pgRNAs can robustly suppress viral spread in higher organisms (*Nicotiana benthamiana*) (A) pgRNAs for RfxCas13d were designed to target pairs of sequences in the tobacco mosaic virus (TMV) variant replicon (TRBO-GFP) genome (left) with target sequences for monovalent (g; black) and polyvalent (pg; red) gRNAs labeled with arrows. (right) After infiltration of the replicon DNA and transcription, the (+) ssRNA virus will infectiously spread cell to cell in the leaf, the extent to which can be tracked by expression of a reporter protein (GFP). Viral spread is inhibited by TRBO-GFP-targeting RfxCas13d RNPs, providing a quantitative assay for antiviral activity by different gRNA designs. MP: movement protein. GFP: green fluorescent protein. (B) Pairs of targets in the TRBO-GFP for the different pgRNAs had up to 30% (6 nt out of 23) divergence between sequences. The sequences of the pgRNAs are optimized for activity at both targets, even with predicted mispairings and non-canonical base pairings with each target. (right) Predicted activity for gRNAs and pgRNAs at each of their paired target sites. (C) Leaves of *N. benthamiana* were infiltrated with a suspension of *A. tumefaciens* harboring plasmids for the transient expression of RfxCas13d; one or two gRNAs (pgRNA, its two “monovalent” counterpart gRNAs, or a non-targeting (NT) gRNA, for example); and an expression cassette for replication-competent TRBO-GFP. Representative images of leaves illuminated under UV light three days after infiltration show the extent of viral spread by GFP expression. To better visualize differences in viral spread between leaves with different gRNAs, image brightness was increased +20% and image contrast increased by +40%. See Figure S4 for additional and unmodified leaf images. Viral spread is suppressed by Cas13 RNPs with gRNAs and strongly by Cas13 RNPs with pgRNAs, but not Cas13 RNPs with a non-targeting (NT) gRNA. In plants treated with pgRNAs, there is some discoloration at the site of agro-infiltration that is distinct from viral GFP. (D and E) Quantitative reverse-transcription PCR (qRT-PCR) of leaf RNA after transient expression demonstrates that pgRNAs successfully inhibit viral spread in a higher organism better than their perfectly-complementary monovalent counterparts, at least as well as multiplexed monovalent gRNAs, and even better as multiplexed pgRNAs— reducing viral RNA levels by >99.5%. Note logarithmic scale on yaxis. Numbers above data points (N = 4 leaves each) are values of mean reduction of viral RNA. Horizontal bars indicate results of two-sided T-test of most relevant comparisons, with pvalue above; see Table S7: RT-qPCR of GFP mRNA in N. bethamiana after transient expression of TRBO-GFP; RfxCas13d; and gRNAs, pgRNAs, or non-targeting (NT) gRNAs for full results of all T-test comparisons. ∗pvalue < 0.05; ∗∗pvalue < 0.005; ∗∗∗pvalue < 0.0005; ns pvalue > 0.05 (not significant). dCas13d: Catalytically inactive RfxCas13dmutant.

To test our hypothesis that pgRNAs targeting multiple viral sites simultaneously would inhibit viral propagation *in vivo* better than their perfectly-complementary-but-monovalent counterparts, we designed pgRNAs for RfxCas13d to target pairs of protospacers found in the tobacco mosaic virus (TMV) (Table S5: RfxCas13d pgRNAs for TRBO-GFP and Associated Properties of the pgRNAs and Table S6: RfxCas13d gRNAs and pgRNAs used in *N. bethamiana*) and infected *N. benthamiana* with a TMV replicon (TRBO-GFP) via *Agrobacterium tumefaciens-*mediated transformation into its leaves (Figures 2A and 2B) (Lindbo, 2007; Mahas et al., 2019). The TRBO-GFP replicon, which has previously been used as a model for viral infection in plants to validate CRISPR-based antiviral biotechnologies (Mahas et al., 2019), contains an expression cassette for a modified TMV under the control of a strong constitutive 35S promoter; after transcription, the replication-competent (+) ssRNA virus can then spread cell-to-cell within the leaf as an uncontrolled infectious agent. Here, the TMV coat protein gene in the TRBO-GFP replicon had been replaced with a green fluorescent protein (GFP) gene that allows viral spread to be visually tracked (Figures 2A, 2C, and S4) and that we use to as a reporter to quantify overall viral RNA levels in the leaves (Figures 2D, 2E and Table S7: RT-qPCR of GFP mRNA in N. bethamiana after transient expression of TRBO-GFP; RfxCas13d; and gRNAs, pgRNAs, or non-targeting (NT) gRNAs). At the time of introduction of the TRBO-GFP replicon into the leaves, we also introduce transfer DNAs (T-DNAs) for transient expression of RfxCas13d via *A. tumefaciens*-mediated transformation and T-DNAs to express either one or two multiplexed gRNAs or pgRNAs (Figures 2A and 2B). The gRNAs and pgRNAs were targeted to the viral replicase gene or movement protein (MP) gene, not the GFP, and designed to avoid the *N. benthamiana* transcriptome by ensuring that each pgRNA contains at least 8 or 10 mismatches (<57–65% complementarity) with all sequenced *N. benthamiana* RNA transcripts (transcriptome assembly v5 (Nakasugi et al., 2014)) (Table S5: RfxCas13d pgRNAs for TRBO-GFP and Associated Properties of the pgRNAs) so the possibility of interaction with host RNA remains unlikely and, even if present, transient. We also performed an additional search for off-targets using a different transcriptome assembly (GIUP: TSA: *N. benthamiana*, transcriptome shotgun assembly) and again, could only find off-targets with >8 or even 10 mismatches for the 3 pgRNAs tested. We otherwise found no evidence of disruption of “off-target” cellular RNA levels when introducing the RfxCas13d and pgRNAs into the plants (Figure S6).

After three days, plants expressing one of six different monovalent gRNAs showed viral RNA levels in their leaves reduced in general to approximately 5–25% of plants expressing Cas13 and either a non-targeting gRNA (gRNA-NT) or no gRNA (Figures 2C–2E). While concerns have been raised regarding the potential toxicity of long-term expression of Cas13d as a result of “collateral activity” (see below) (Tong et al., 2022), Cas13d-treated plants appeared phenotypically healthy over the course of the experiments (Figure 2C). Plants expressing a single monovalent gRNAs exhibited in general less viral replication suppression than those expressing a single pgRNA, which were able to robustly suppress viral spread (Figure 2C) and viral gene expression by >97% relative to GFP mRNA levels in plants expressing a gRNA-NT (Figures 2D, 2E, S4, and Table S7: RT-qPCR of GFP mRNA in N. bethamiana after transient expression of TRBO-GFP; RfxCas13d; and gRNAs, pgRNAs, or non-targeting (NT) gRNAs). We note that one particularly high-performing “monovalent” gRNA (gRNA TRBO.np5360 or gD) was also found to have significant predicted “polyvalent” behavior at its paired site even prior to sequence optimization (Figure 2B), meaning that this spacer sequence would be have been algorithmically rejected from other gRNA design approaches where predicted activity at multiple sites is not allowed.

The improved performance by the pgRNAs relative to their monovalent counterparts is remarkable considering that the pgRNA spacer sequence contains up to three or four imperfectly (noncanonically) complementary or mis-paired nucleotides with each of its two targets (Figure 2B). One exceptional pgRNA (pgEF) reduced viral RNA levels across the leaf (to 0.28%) 30- to 40--fold relative to its monovalent counterparts (to 11.4 and 9.3% for gE and gF, respectively) despite lacking the complementarity with either target that the monovalent gRNAs possess. These results would suggest that the “polyvalency” or ability for a single CRISPR effector to recognize multiple targets on a virus of pgRNAs can more than compensate for any potential reductions in activity as a result of mispairing or “mismatch penalties” at those targets *in vivo* in suppressing an active viral infection.

### RfxCas13d with pgRNAs perform better than RfxCas13d with multiplexed (perfectly-complementary) “monovalent” gRNAs, and multiplexing pgRNAs (targeting four sites with only two guide RNAs) improves viral suppression further

The plants expressing the pgRNA exhibited reduced viral levels (often <2% viral RNA levels relative to non-targeting controls) that were at least similar to and often better than those plants undergoing multiplexed expression of two of their (perfectly-complementary) “monovalent” counterparts (1.7%–7.2% viral RNA levels relative to non-targeting controls). Multiplexed expression of two sets of the three pgRNAs—together targeting four viral targets simultaneously with two guides—further reduced viral RNA levels by an order of magnitude, in two cases to 0.33–0.6% viral RNA in the leaves compared to plants expressing the non-targeting gRNA. A third multiplexed pgRNA set “only” reduced viral RNA levels to 5%, levels equivalent to multiplexed monovalent gRNAs, which may have been a result of a predicted partial base pairing interaction between the two pgRNAs when both are expressed simultaneously (Figure S5). The antiviral effect of pgRNAs is mediated by the targeted ssRNAse activity of Cas13d (Figure 2D), although treatments with a catalytically inactive Cas13d variant (dCas13d) were found to exhibit a modest (10–40%) reduction of viral RNA levels in *N. benthamiana* through some as-yet-unknown mechanism, perhaps related to a similarly modest silencing effect previously reported in *N. bethanamiana* when gRNAs were expressed in the absence of any Cas enzymes (Sharma et al., 2022). These results demonstrate that catalytically active Cas13 is necessary for the strong antiviral effect of pgRNAs. The significant inhibition by pgRNAs of viral propagation and spread during infection therefore suggests that polyvalent targeting of viruses using pgRNAs might represent a superior paradigm for gRNA design in CRISPR antiviral applications and further highlights the potential for CRISPR effectors as viral prophylactic and treatments in plants and other organisms.

We also note that in practice and particularly in non-transgenic organisms or cell lines, even generating arrays for multiplexed expression of gRNAs targeting four distinct sites can be non-trivially challenging for any CRISPR effector, including Cas13 (Liao et al., 2019). The ability to use pgRNAs in a way that results in an operational multiplexing where four sites are simultaneously targeted using only two gRNAs represents a significant advance in that regard.

### pgRNAs can be engineered to stimulate “collateral activity” by Cas13 for *in vitro* viral detection applications at viral sequences diverging by up to 25%

Cas13 exhibits both a specific “cis cleavage” ssRNAse activity on binding to its targeted RNA as well as nonspecific ssRNAse activity “*trans* cleavage” activity or “collateral activity” that occurs after target recognition and conformational change (Nalefski et al., 2021; Zhang et al., 2018). This collateral activity is observed in different classes of Cas13 effectors, and a conserved mechanism across these different effectors is that target recognition/RNA-base-pairing and activation of RNAse activity are tightly coupled (O'Connell, 2019). This target-activated “collateral activity” has been used for applications in viral diagnostics such as SHERLOCK (Figure 3A) (Ackerman et al., 2020; Fozouni et al., 2021; Freije et al., 2019), including in a diagnostic assay for SARS-CoV-2 (Figure 3C), the (+) ssRNA coronavirus responsible for the COVID19 respiratory infection. In viral detection systems using CRISPR effectors like SHERLOCK, which typically uses Cas13a from *Leptotrichia wadei* (LwaCas13a), it has been found that multiplexed use of multiple gRNAs improves viral detection sensitivity (Fozouni et al., 2021; Nalefski et al., 2021), using up to 20 separate gRNAs. Although recognizing additional viral sites would be expected to increase robustness to genetic variation across strain variants or allow detection of multiple viruses within a family, inclusion of so many additional gRNAs may result in increased complexity in assembly and/or operation of the assay, as well as potentially increased cost per reaction. Therefore, we also sought to determine whether pgRNAs could be used for these *in vitro* applications to trigger collateral activity at multiple viral targets, simultaneously, with potentially fewer components.

LwaCas13a is the Cas13 used in the SARS-CoV-2 SHERLOCK detection assay that received emergency use authorization (EUA) by the U.S. Food and Drug Administration, and purified LwaCas13a enzyme is also commercially available, but LwaCas13a has not been as exhaustively characterized as RfxCas13d with regards to tolerance to mismatches. We hypothesized that if the major contribution to activating Cas13 RNAse activity was the stability of base-pairing between gRNA spacer and target, we could use the mismatch sensitivities for RfxCas13d to provide at least a starting point or basis for designing pgRNAs for LwaCas13a for viral detection applications. This proved to be the case: despite SHERLOCK and the activation of collateral activity having been reported to be sensitive to single-nucleotide polymorphisms in their targets, (Ackerman et al., 2020; Gootenberg et al., 2018), we found that even when minimizing predicted “mismatch penalties” according to design rules for RfxCas13d we could engineer single pgRNAs that could successfully trigger LwaCas13a collateral activity at multiple synthetic and SARS-CoV-2 derived RNA targets that diverged by up to 25% (6 out of 23 nt) (Figures 3B and 3D), and which could even exhibit collateral activity at targets with up to 4 nt mismatches with the gRNA spacers (Figure 3B). This polyvalently-triggered collateral activity was specific to the engineered pgRNAs: regular (perfectly matched) “monovalent” gRNAs exhibited effectively no cross-reactivity *in vitro* at paired sites with such high sequence divergence (Figure 3D). These results suggest that, at least as a starting point and at least *in vitro*, pgRNAs for LwaCas13a could be designed to trigger collateral activity at two specific targeted sites, even when using design rules for RfxCas13d.

To assess whether pgRNAs might be suitable for *in vitro* viral diagnostics, we generated a series of 23 pgRNAs with high predicted activity (using RfxCas13d design rules) at 15 target pairs found in SARS-CoV-2 (Table S8: RfxCas13d pgRNA Target Pairs and Sequence Conservation for SARS-CoV-2, Table S9: RfxCas13d pgRNAs for SARS-CoV-2 and Associated Properties of the pgRNAs, Table S10: RfxCas13d pgRNAs for SARS-CoV-2 used in Collateral Activity assays, and Figure S7), then screened their collateral activity in the presence of their SARS-CoV-2 RNA targets and compared those results with the combined activity their perfectly matched monovalent gRNA counterparts (30 separate gRNAs). We found that each of the pgRNAs tested exhibited collateral activity at levels similar to or higher than their combined monovalent gRNA counterparts with both targets present in the same sample (Table S11: Results of SARS-CoV-2 collateral activity assays), and no off-site collateral activity was detected above background in the presence of non-targeted RNA sequences, universal human reference RNA (10 human cell lines; Thermo Fisher Scientific), or human lung total RNA (Thermo Fisher Scientific) (3 μg RNA). We then assessed their limits of detection (LoD) in an SHERLOCK-type assay using LwaCas13a and the best-performing pgRNAs, and found that Cas13 with single pgRNAs (recognizing two sites) or two pgRNAs (recognizing four) could robustly generate detectable signals in samples initially containing 40 cp/uL heat-inactivated SARS-CoV-2 (clinically relevant LoD for SARS-CoV-2 is often considered to be 1000 cp/uL) (Fozouni et al., 2021) (Figure 3E), performing as well as their monovalent counterparts and even some multiplexed monovalent gRNAs in this assay.

These results suggest that pgRNAs are suitable for *in vitro* viral detection applications of multiple viral targets with fewer components than multiplexed detection using monovalent gRNAs, and therefore provide potentially simpler assembly or operation and lower cost per reaction. However, we do note that, like in PCR-based diagnostic assays, we found that a major determinant for diagnostic specificity in the SHERLOCK-type assays was the set of primers used to amplify the viral nucleic acids prior to their mixture with Cas13. Therefore, we do not expect the use of pgRNAs that have been engineered to recognize two specific viral targets will increase the potential for “false positives” in an assay for a specific virus of interest, provided primers for viral nucleic acid amplification are designed properly. Rather, that Cas13-based viral detection assays using pgRNAs can be triggered by two specific viral sequences that can diverge by up to 25% (with mismatches at 6 out of 23 nt) implies their use compared to perfectly-matched gRNAs might increase assay robustness with regards to genetic variation across clinical variants, particularly under conditions where it might not be likely for variants to have so many mutations at both targeted sites simultaneously. For example, a variant with a large indel at one site might not be detectable in an CRISPR-based viral detection assay using a monovalent gRNA targeted to that site, but it is less likely that a variant will have large indels at all of the multiple sites recognized by a single pgRNA.

Additionally, the design principles of pgRNAs could also be applied to develop broad-spectrum Cas13-based viral diagnostics that are tolerant to polymorphisms across a viral family (Lin et al., 2021), e.g., for viral surveillance of emerging or zoonotic pathogens. In those applications, the advantages of CRISPR-based viral diagnostics, such as isothermal detection, would be particularly important for remote or resource-poor settings compared to PCR based diagnostics.

### pgRNAs can be engineered to stimulate Cas9 dsDNA cleavage activity *ex vivo* at target pairs in DNA that diverge by up to 40%

**Figure 4:**
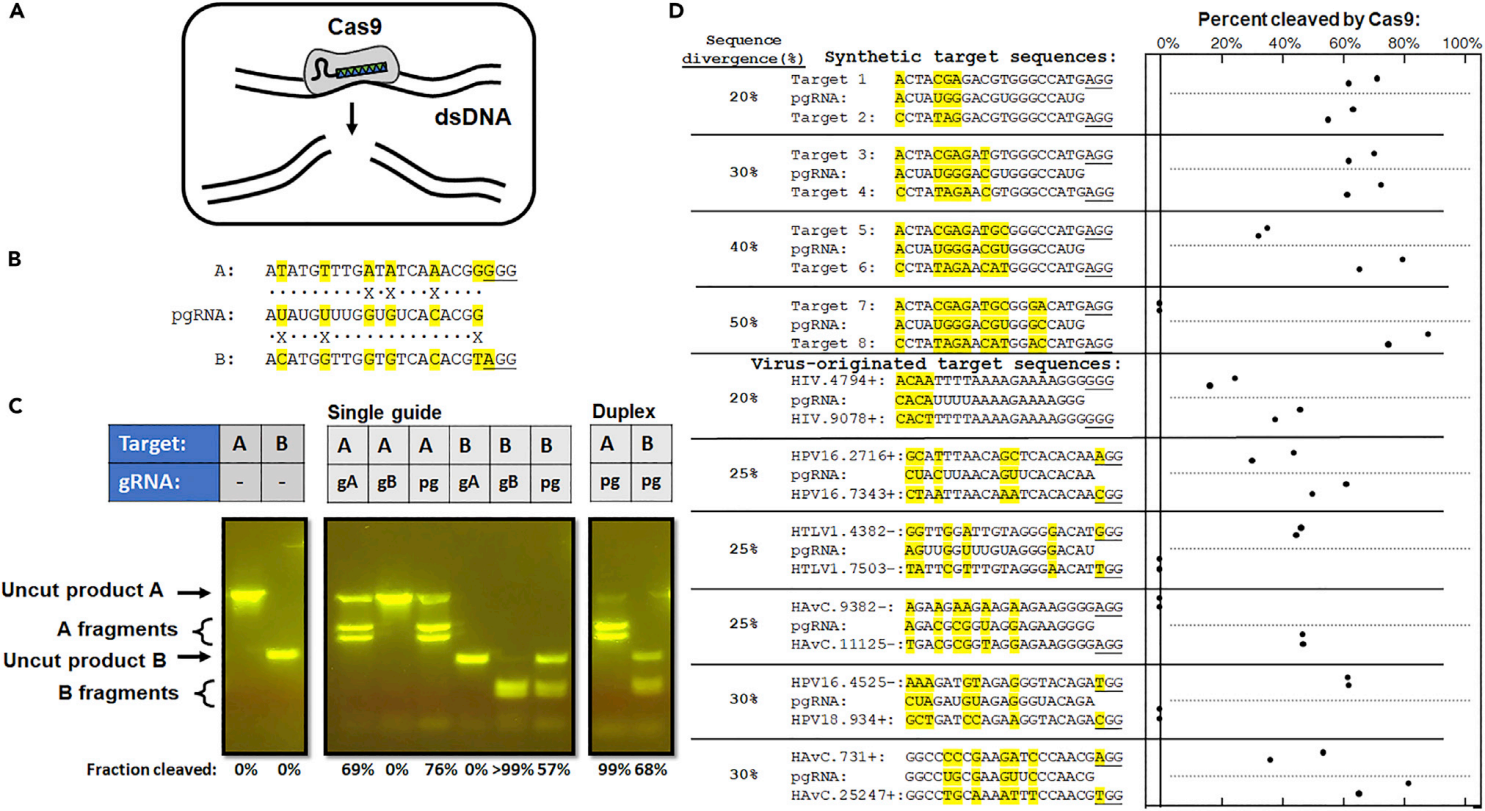
pgRNAs can be engineered to stimulate Cas9 dsDNA cleavage activity at divergent target pairs *ex vivo* (A) Cas9 recognizes and cleaves dsDNA. (B) A pgRNA (pg) and its two “monovalent” counterpart gRNAs (gA and gB) for Cas9 from *S. pyogenes* was designed to target two sequences that differ by 6 of the 20 nt (30%) in their protospacer region, and 1 out of 3 within their protospacer adjacent motif (PAM) region (underlined). (C) Cas9 with monovalent guides exhibit no cross-reactivity at homeologous sites, while Cas9 with a pgRNA exhibits robust cleavage activity at both sites. pgRNA activity is enhanced with a crRNA:tracrRNA duplex compared to a chimeric “single guide” RNA. (D) pgRNAs could be generated for SpyCas9 to exhibit robust cleavage activity *ex vivo* at pairs of synthetic targets (upper) and target sequences originally found in DNA viruses or retroviruses (lower) with sequences diverging by up to 40%. HIV, HIVtype 1; HPV16, Human papillomavirus type 16; HPV18, Human papillomavirus type 18; HTLV1, Human T-lymphotropic virus 1; HAvC, Human Adenovirus C.

Last, we sought to determine whether the design principles we use for pgRNAs in Cas13 could be applied to gRNAs for other types of CRISPR effectors like the Cas9 effector from *Streptococcus pyogenes* (SpyCas9), which recognizes and introduces double-strand breaks into dsDNA targets (Figure 4A) (Lee, 2019). We designed pgRNAs to target homeologous pairs of DNA sequences with sequence divergence up to 50%, that is, differing at up to 10 of the 20 bp sites in the SpyCas9 protospacers (Table S12: Cas9 gRNAs, pgRNAs, and targets for *ex vivo* validation of the pgRNA design protocol) and measured the cleavage activity of purified Cas9 RNPs at those sites *ex vivo* (Figures 4B–4D). Some of these sequences were identified from DNA viruses or retroviruses as potentially-targetable pairs, while others were designed to test the maximum extent of “polyvalent” activation (Figure 4D). As with Cas13, SpyCas9 with “monovalent” (perfectly matched) gRNAs exhibited no cross-reactivity at paired sites with such high sequence divergence (Figures 4B, 4C, and S8), while SpyCas9 RNPs with pgRNAs could consistently cleave both targets even when paired sequences diverged by up to 40% (Figures 4 and S9). In cases where the pgRNA only exhibited activity at one target, those targets could still possess up to 5 mismatches between the pgRNA spacer and the protospacer. In addition, we found that including a leading 5′- rG on the spacer, a condition thought to result in greater specificity in CRISPR activity for gene editing applications (Kim et al., 2014), consequently reduced pgRNA activity at both sites (Figure S10). Hence, by optimizing the tolerance for mismatches between the spacer sequence and targeted sites, we show that pgRNAs can also be engineered to promote high levels of SpyCas9 cleavage activity at multiple targeted DNA sequences simultaneously *ex vivo*. These results demonstrate that our protocol of modifying gRNAs to minimize “mismatch penalties”—the effects of position- and mismatch-specific reductions in activity that have been rigorously characterized for several Cas9 variants, Cas12 variants, and RfxCas13 (Figure S3)—at multiple sites allows us to engineer intentional “polyvalency” that can be applied in diverse CRISPR systems, targeting both DNA and RNA.

## Discussion

The CRISPR effector proteins used in biotechnological applications were originally found in bacteria and archaea as an antiviral mechanism to degrade foreign DNA and RNA (Shmakov et al., 2017), and so some tolerance to sequence variation in their targets is likely beneficial for this purpose. In gene editing applications, significant efforts are made to limit the natural tolerance of CRISPR effectors for nucleic acids with imperfect complementarity to their gRNAs in order to prevent degradation and mutation at unintended or “off-target” sites; here we exploit those tolerances to engineer gRNAs that are optimized to promote activity at multiple viral target sites, simultaneously. We show that the polyvalent targeting of viruses by single engineered gRNAs—optimized based on the CRISPR effector’s natural position- and sequence-determined tolerance for mismatches for activity at the homeologous target pairs that are abundant in viral genomes—(1) can drive robust CRISPR activity at specific targeted pairs simultaneously *in vitro/ex vivo*, (2) can exhibit stronger viral suppression despite mismatches with viral targets during infection of a higher organism relative even to perfectly-complementary “monovalent” targeting across organ and tissue scales, and (3) may in fact be optimal for applications of CRISPR antiviral diagnostics, prophylactics, and therapeutics. pgRNAs can also be synergistically integrated into strategies currently used in CRISPR antivirals to suppress mutational escape, such as gRNA multiplexing and targeting regions of high sequence conservation (or pairs of targets with high sequence conservation) (Figure S7) and to engineer viral diagnostics that are robust to potential clinical polymorphisms.

We emphasize that pgRNAs are engineered to target two specific viral sites, not to generally allow their CRISPR effector to be more prone to “off-target activity” or to tolerate more mismatches than any other gRNA or CRISPR effector using conventional gRNA designs. Both Cas13d and Cas9 have well-characterized natural tolerances to mismatches between their gRNA spacer sequence and target that we exploit for the specific targeting of two selected viral sites. For example, as mentioned above, Cas13d requires at least 18 nts of complementarity (fewer than 5 mismatches for a 23 nt spacer) to its target for stable binding and target cleavage to occur (Zhang et al., 2018). In Figures 3C and 3D, the pairs of target sequence each differ from each other by 5 or 6 nucleotides, but the engineered pgRNAs only have 3 mismatches with each and/or non-canonical base-pairing that are still stable (rG-rU base-pairs, for example) and are activated at both sites after optimization. Conversely, we see that their respective “monovalent” gRNAs exhibit no cross-reactivity at the paired sites, since the “monovalent” spacers each have at least 5 or 6 mismatches with their paired sequence. Consequentially, to suppress any potential off-target activity triggered by host nucleic acids, as part of our algorithmic design process, any potential pgRNA is rejected unless it has at least 8 mismatches with any known host transcripts (for Cas13). While of course every gRNA sequence should be evaluated for off-target interactions with the host genome or transcriptome, we expect the profile of off-target effects for pgRNAs to be like those of conventionally designed gRNAs that are similarly selected to avoid interaction with the host genome or transcriptome.

It is remarkable and perhaps counterintuitive that despite multiple mismatches and/or non-canonical base pairings with any of their viral targets, pgRNAs with Cas13 could significantly outperform their monovalent counterparts that have perfectly complementary to viral sequences—in one case by decreasing viral RNA across a tissue by 30- to 40--fold relative to the monovalent gRNAs. Single pgRNas were also shown they can outperform their monovalent gRNAs even when those pairs of monovalent gRNAs were multiplexed. Though further investigation is necessary, we propose that this improvement of pgRNAs in ability to suppress viral propagation may be related to the kinetics of CRISPR effector recognition of a rapidly replicating RNA virus, a property that may be more important in antiviral applications than for other applications of CRISPR biotechnologies. That is, when performing multiplexed targeting with N distinct “monovalent” gRNAs, the amount of transfected CRISPR effector is likely limiting, and so this multiplexing can be thought to effectively reduce the number of effectors per target by 1/N. Although multiplexed targeting likely helps in preventing viral mutagenic escape, this effective reduction in effector concentration per target may have critical implications in the rate that each CRISPR effector can find and degrade their viral target on a rapidly replicating RNA virus in a eukaryotic cell. In contrast, a CRISPR effector with single pgRNA that can recognize N viral sites while still maintaining high activity at each site now has N-fold the number of viral targets it can potentially recognize, which would provide an enhancement in the rate of viral recognition per effector even over effectors with perfectly matched “monovalent” gRNAs that can only recognize a single viral site. It is perhaps this influence on the kinetics of effector recognition and degradation—which would potentially be less critical in gene editing applications or even in applications of Cas13-mediated “knock-down” of mRNA transcripts from a discrete set of chromosomal genes than for a viral target—that results in improved viral suppression. Based on our results *in planta*, it appears the “polyvalency” of pgRNAs can compensate for any potential reduction of activity at any of its target sites in a way that improves their antiviral activity with Cas13 over their perfectly-complementary monovalent counterparts.

Although here we used Cas13 in whole *N. benthamiana* plants as a proof-of-concept to demonstrate the superiority of pgRNAs in antiviral applications of CRISPR, work in our laboratory is ongoing to determine whether pgRNAs can also be effective against human viruses in human cell lines. The prospect of CRISPR antiviral therapeutics is promising because they can be rapidly reconfigured in the face of emerging viral pathogens and could potentially be delivered as a transient antiviral agent during an acute infection (Blanchard et al., 2021); however, there still remain significant barriers to clinical translation that must be considered, including with regards to any potential off-target or host-targeting effects, and in particular for DNA-targeting CRISPR enzymes like Cas9. Although the prospect of interactions with the host genome or transcriptome is bioinformatically minimized with our pgRNA design algorithm, potential off-target effects will need to be experimentally evaluated very carefully for each potential viral target and (p)gRNA sequence to ensure their safety. Another potential concern has been raised with regards to potential toxicity that can occur as a result of Cas13’s collateral activity (Özcan et al., 2021; Tong et al., 2022), although in our study plants appeared phenotypically healthy. This toxicity has been observed in animal cell lines and transgenic animals under conditions where both Cas13 and its target are highly expressed during for targeted RNA knock-down by Cas13 for long periods of time, conditions not likely to be necessary for antiviral applications. It remains to be seen whether or not Cas13’s collateral activity is necessary for its antiviral effects *in vivo* but, if not, engineered Cas13 variants with targeted activity but minimized collateral activity (Tong et al., 2022) or alternative RNA-targeting CRISPR effector Cas7-11 that does not exhibit collateral activity (Özcan et al., 2021) could provide potentially safer opportunities. In addition, although previous studies in human cell lines did not find evidence of elevated mutation rates in CRISPR-targeted viruses (Freije et al., 2019), our laboratory is currently evaluating “long-term” effects of CRISPR antivirals on the mutation rate of viral targets, as this will be another critical consideration; we suspect that use of pgRNAs can further help suppress the potential for viral mutagenic escape. Regardless of their potential as a human therapeutic, our work also suggests that CRISPR antivirals in plants might itself represent a powerful application of these biotechnologies, as these applications circumvent many of the clinical barriers to entry that human antivirals will require with regards to safety. Plant viruses account for about 50% of all plant diseases, are associated with famine events, and cost over $30 billion dollars a year in crop losses, with limited potential for treatments or prophylactics (Nicaise, 2014). CRISPR biotechnologies may represent a powerful approach in that regard, particularly as we report that pgRNAs appear to be so effective in limiting viral spread in plants.

pgRNAs may also be a useful tool for functional virology, as Cas13-mediated knock-down or degradation of targeted viral mRNAs may be more challenging with a rapidly proliferating virus with many replicates, when compared to knock-down of gene expression during typical transcription from a eukaryotic cell. Furthermore, while pgRNAs were designed with antiviral applications in mind, we showed our design principles also work for both RNA- and DNA-targeting CRISPR effectors, and so pgRNAs could in principle be designed for “polyvalent” genome editing using CRISPR, particularly for knock-out of members of large orthologous families of genes where coverage across the entire family that might be difficult if using traditional multiplexed gene editing approaches with large numbers of “monovalent” guides rather than pgRNAs.

### Limitations of the study

In this proof-of-concept, we demonstrated that pgRNAs can provide superior viral elimination compared to conventionally-designed gRNAs at organ/tissue scales using a plant and a plant virus; it remains to be confirmed that pgRNAs will provide a similar antiviral enhancement in animals, though because multiplexed targeting improves antiviral activity in animal (human) cell lines there is reason to believe pgRNAs should do so in animals as well. In addition, although polyvalent targeting of divergent was demonstrated *in vitro* with Cas9 with pgRNAs, we have not yet demonstrated that pgRNAs will increase antiviral potential of CRISPR biotechnologies against a DNA virus. Finally, we stress that while, mechanistically, there is no reason to believe that pgRNAs should be more prone to “off-target” activity or interaction with host nucleic acids than any other conventionally-designed gRNAs, off-target activity should be determined for each gRNA in each future application.

## STAR★Methods

### Key resources table

**Table:** 

REAGENT or RESOURCE	SOURCE	IDENTIFIER
**Bacterial and virus strains**		

*Escherichia coli* TOP10	Thermo Fisher	Cat#C404010
*Escherichia coli* NEB10B	New England Biolabs	Cat#C3019H
*Agrobacterium tumefaciens* GV3101	Gold Biotechnology	Cat#CC-207

**Chemicals, peptides, and recombinant proteins**		

Sodium carbonate	Fluka	Cat#71350
EnGen RNA synthesis kit	New England Biolabs	Cat#E3322V
Monarch RNA cleanup kit	New England Biolabs	Cat#T2040S
Universal Human Reference RNA	Invitrogen	Cat#QS0639
Human Lung Total RNA	Invitrogen	Cat#AM7968
Monarch Plasmid Miniprep Kit	New England Biolabs	Cat#T1010S
Taq 2X Master Mix	New England Biolabs	Cat#M0270L
RNeasy Plant Mini Kit	Qiagen	Cat#74904
DNAse I	Ambion	Cat#AM2222
iTaq PowerUP SYBR Green 2x Master Mix	Applied Biosystems	Cat#A25741
Acetosyringone	Sigma Aldrich	Cat#D134406G
Magnesium chloride hexahydrage	MP Biomedicals LLC	Cat#195304
MgCl_2_ (1 M)	Invitrogen	Cat#AM9530G
MOPS sodium salt	Gold Biotechnology	Cat#M-791
Q5 high fidelity DNA polymerase	New England Biolabs	Cat#M0491S
DpnI	New England Biolabs	Cat#R0176S
XhoI	New England Biolabs	Cat#R0146S
XbaI	New England Biolabs	Cat#R0145S
RNase Inhibitor, Murine	New England Biolabs	Cat#M0314S
T7 RNA polymerase	New England Biolabs	Cat#M0251S
Ribonucleotide Solution Mix	New England Biolabs	Cat#N0466S
SuperScript™ III First-Strand Synthesis System	Invitrogen	Cat#18080051
*Leptotrichia wadeii* Cas13a (LwaCas13a)	MCLAB	Cat#CAS13a-100
Cas9	New England Biolabs	Cat#M0386S
NEBuffer 3.1	New England Biolabs	Cat#B7203
Proteinase K, Molecular Biology Grade	New England Biolabs	Cat#P8107S
RNAse Alert	Integrated DNA Technologies	Cat#11-02-01-02
HEPES (1 M)	Gibco	Cat#15630080
High-Capacity cDNA Reverse Transcription Kit	Applied Biosystems	Cat#4368814
Kanamycin sodium salt	Thermo Fisher	Cat#611770250
Gentamycin sulfate	Thermo Fisher	Cat#613980010
Rifampin	Thermo Fisher	Cat#J60836.06
Ampicillin sodium salt	Thermo Fisher	Cat#611770250
LB Media	Sigma Aldrich	Cat#L3022
Agar	Sigma Aldrich	Cat#A1296
Gel Loading Dye	New England Biolabs	Cat#B7024S
SYBR™ Green I Nucleic Acid Gel Stain - 10,000X concentrate in DMSO	Thermo Fisher	Cat#S7563
Heat-inactivated SARS-CoV-2 RNA from respiratory specimens	BEI Resources	Cat#VR-1986HK (ATCC)

**Critical commercial assays**		

Sherlock CRISPR SARS-CoV-2 Kit	Integrated DNA Technologies	Cat#10006968

**Deposited data**		

Viral genomic sequences	NCBI Virus	see Tables S1 and S13
Human genome sequence	NCBI	Genome Reference Consortium Human Build 38 (GRCh38)
Human transcriptome sequence sequence	NCBI	Genome Reference Consortium Human Build 38 (GRCh38)
*Nicotiana benthamiana* transcriptome sequenes	Nakasugi et al., 2014	transcriptome assembly v5

**Experimental models: organisms/strains**		

Model organism: *Nicotiana benthamiana*	Dr. Million Tadege, Oklahoma State University	Zhang et al., 2014

**Oligonucleotides**		

Oligonucleotides	Integrated DNA Technologies	see Table S13
Alt-R® CRISPR-Cas9 tracrRNA	Integrated DNA Technologies	Cat#1072532
Alt-R® CRISPR-Cas9 crRNA	Integrated DNA Technologies	see Table S13
Gene Fragments	Twist Bioscience	see Table S13

**Recombinant DNA**		

Plasmids	Addgene	see Table S13

**Software and algorithms**		

MATLAB with Bioinformatics Toolbox	MathWorks (Natick, MA)	R2018a+
Original code	This work	https://www.github.com/ejosephslab/pgrna (https://doi.org/10.5281/zenodo.7126522)
Python	https://www.anaconda.com/products/distribution	v3.1
BLAST 2.8.1+	NCBI	Camacho et al., 2009
RNAfold	Lorenz et al., 2011	RNAfold installer https://www.tbi.univie.ac.at/RNA/
Biopython	Cocket al., 2009	https://biopython.org/wiki/Packages

### Resource availability

#### Lead contact

Further information and requests for resources and reagents should be directed to and will be fulfilled by the lead contact, Eric Josephs (eric.josephs@uncg.edu).

#### Materials availability

Plasmids generated during this are available on request to the lead contact.

### Experimental model and subject details

#### Nicotiana benthamiana

Seeds for *N. benthamiana* were obtained from Dr. Million Tadege at Oklahoma State University (Zhang et al., 2014). *N. benthamiana* plants grown under long-day conditions (16 h light, 8 h dark at 24°C).

#### Agrobacterium tumefaciens

*A. tumefaciens* strain GV3101 were grown on LB (10 g/L tryptone, 5 g/L yeast extract, 10 g/L NaCl; pH 7)-agar plates containing antibiotics appropriate for the plasmids propagated by the bacteria at 28°C. Single colonies were grown overnight at 28°C in LB containing antibiotics appropriate for the plasmids propagated by the bacteria prior to agro-infiltration of plants.

#### Escherichia coli

*E. coli* strain TOP10 (Thermo Fisher) were grown on LB-agar plates containing antibiotics appropriate for the plasmids propagated by the bacteria at 30 or 37°C. Single colonies were grown overnight at 30°C or 37°C in LB media containing antibiotics appropriate for the plasmids propagated by the bacteria.

### Method details

#### Design of polyvalent guide RNAs

The pgRNA design algorithm was implemented in MATLAB (MathWorks, Inc.) with the Bioinformatics toolbox installed or Python 3.1 with the biopython package (Cock et al., 2009) using code written in-house and made available at: https://github.com/ejosephslab/pgrna (https://doi.org/10.5281/zenodo.7126522). Viral genome sequences used to identify targets are listed in Table S13: Oligos, DNA , and Genomic sequences used in this study. To elaborate on each step of the protocol:

Step 1: Estimate activity at different targets (‘protospacers’). “On-target” activity for every potential target in a viral genome is found using sgRNA Designer for Cas9 (Doench et al., 2016; Listgarten et al., 2018) or cas13design for Cas13d (Wessels et al., 2020) software. Only those targets with predicted activity in the top quartile are generally considered as potential pgRNA targets.
Step 2: Identification of Targetable Pairs with high homology. Every potential target is aligned to every other potential target, and pairs with >70% sequence identity (≥14 nt identity for 20 nt Cas9 targets and ≥16 nt identity for 23 nt Cas13d targets) are identified.
Step 3: Optimization of pgRNA activity at pair sequences. For a given target pair, a pgRNA spacer template was generated complementary to the targets, using the location and sequences of the matching targets. Different ‘candidate pgRNA’ spacers were generated with all four potential nucleotides (rA, rU, rC, rG) at each of the sites of sequence divergence between the target pairs, *i.e.* 4^n^ candidates for target pairs with n differences between sequence. A mismatch penalty (CFD score, see below) between the candidates and each of the target pairs was calculated using the multiplicative approach (*i.e.*, Figure 1C right). Those with activity at both sites predicted to remain in the top quartile (or other threshold) for both were kept for further evaluation. Candidate pgRNAs with homopolymer repeats (≥4 consecutive ‘rU’ or ≥5 consecutive ‘rG’, ‘rC’, or ‘rA’) were removed. Those with GC% <30% or >70% were also removed from consideration. For RfxCas13d, the respective ‘direct repeat’ sequence for each crRNA (5′-ACCCCUACCAACUGGUCGGGGUUUGAAAC-3′) sequence was appended 5′- to their pgRNA candidate spacers and the pgRNA secondary structures evaluated using the RNAfold function from MATLAB’s Bioinformatic Toolbox or Vienna RNA (Lorenz et al., 2011) If the secondary structure of the direct repeat was perturbed by presence of the candidate spacer from its canonical structure, it was removed from consideration, as were those with secondary structure free energy in the spacer region lower than −5 kcal/mol.
Step 4: Estimate activity at potential host off-targets. Candidate pgRNA spacers are aligned to the host genome or transcriptome—in this manuscript for *N. benthamiana* we used transcriptome assembly v5 (Nakasugi et al., 2014)—using a local nucleotide BLAST optimized for short sequences <30 nt (blastn-short). The region surrounding each hits to the human genome or transcriptome, to a total of 23 nt (the 23 nt protospacer for Cas13d and 20 nt protospacer + 3 nt PAM for Cas9), were evaluated for a mismatch penalty score with its respective pgRNA candidates and, for Cas9, the presence of PAM. Those with no predicted interaction with the host genome or transcriptome are considered the leading candidates and exclusively used in this work with *N. benthamiana*.
Step 5: Selection of pgRNA based on additional functional criteria. At this stage, the pgRNA candidates have been computationally screened for high activity at multiple viral targets, no predicted activity at host “off-target” sites, and biophysical characteristics that suggest they would retain high overall intracellular CRISPR activity (Doench et al., 2016; Wessels et al., 2020). In principle, the candidates could then be further refined by considering pgRNA targets located within specific genes or regions of interest (ROIs) that may be of clinical or functional significance, or conservation of the targets / viral intolerance to mutations, prior to experimental validation.

#### Calculation of mismatch penalties and predicted CRISPR activities

Estimates of the predicted CRISPR activity at sites not perfectly targeted by the gRNA/pgRNA spacer sequence were generated by calculating the Cutting Frequency Determination (CFD) score (Doench et al., 2014, 2016). To calculate the CFD score, the penalty (relative reduction in CRISPR activities) that result from each site with a mismatch is first drawn from a CFD matrix, the table of position-specific reductions of activity that occur as a result of mispairing between specific nucleotides in the spacer and target. The CFD matrices for CRISPR effector were generated by the Sanjana Laboratory (RfxCas13d (Wessels et al., 2020)) and Doench Laboratory (SpyCas9 (Doench et al., 2016; Doench et al., 2014)), using the data from the “dropout” experiments) using massively parallel screens of gRNA libraries for CRISPR activity, and CFD scoring implemented using publicly available data sets from those labs. The CFD score for a given target and gRNA spacer is the product of the CFD penalties for each mismatch; the position-specific penalties (average overall possible mismatched nucleotides) are summarized in Figure S3. This approach is fast to implement and has been successfully used as a reasonable approximation for CRISPR activity at off-target sites by for a number of different CRISPR effector (Doench et al., 2014; Tycko et al., 2018). In the case of RfxCas13d, penalties were recovered from taking the value of the reported log2(Fold-Change in expression) to the second power, versus a perfectly complementary targeted mRNA reporter in their massively parallel screen for gRNA activity in the presence of mismatches (Wessels et al., 2020). A missing value (rA-rC mismatch at position 15) was interpolated from the penalties of the rA-rC mismatches at positions 14 and 16. In the event of multiple sequential mismatches (two-in-a-row, three-in-a-row, etc.), the position-specific penalties for double- and triple- mismatches were used to calculate the CFD scores at those sites. In the case of Cas9, changes in the relative activity at sites with non-canonical PAM sequences (apart from the canonical PAM sequence d(NGG)) are also included as part of the CFD score.

#### Prevalence of pgRNA target pairs in viral genomes and pgRNA candidates for human-hosted viruses

All complete sequences of all RNA viruses with human, mammal, arthropoda, aves, and higher plant hosts found in the NCBI Reference Sequence database were subjected to a brute force direct (nucleotide-by-nucleotide, no gaps) alignment for each of their 23 nt sequence targets to each other, considering only sequence polymorphisms at the same site. We considered only the (+) strand, as even for (−) and dsRNA viruses these sequences would match the vast majority of mRNA sequences. Only targets lacking polynucleotide repeats (4 consecutive rU’s, rC’s, rG’s, or rA’s) were considered viable targets. Targets derived from different segments or cDNAs of the same viral strain were considered together. In total: arthropoda (1074 viral species), aves (111), mammal (496), higher plant/embrophyta (691), and human (89)-hosted viruses were considered (Table S1: Statistics of Homeologous Cas13 Target Pair (>16/23 or 70% sequence identity) Prevalence in RNA viral genomes, Table S2: Homeologous Cas13 Target Pairs (>16/23 or 70% sequence identity) of Human-hosted RNA viruses, and Table S3: Statistics of pgRNA Candidate Prevalence in human-hosted (+) ssRNA viral genomes and viral RNA transcripts). For human-hosted (+) ssRNA viruses or sequenced viral transcripts (59 in the RefSeq database), candidate pgRNA sequences for RfxCas13d were generated for each target pair found with predicted (monovalent) activity at both sites to be in the top quartile, (Wessels et al., 2020) screened for biophysical compatibility (lacking polynucleotide repeats or significant predicted secondary structure in the spacer), and aligned to Genome Reference Consortium Human Build 38 GRCh38 human reference transcriptome) using a local nucleotide BLAST (Camacho et al., 2009) search optimized for short sequences <30 nt (blastn-short). Only those with no hits (less than 15 nt homology out of 23 nt targets) to the human transcriptome and with predicted activity at both sites to be within the top quartile of all Cas13 activity for targets of that virus were considered viable pgRNA candidates.

#### Construction of RfxCas13d for *in planta* expression

The DNA sequences of the plant codon optimized Cas13d-EGFP with the Cas13d from *R. flavefaciens* (RfxCas13d) flanked by two nuclear localization signal (NLS) was amplified from plasmid pXR001 (Addgene #109049) using Q5 high fidelity of DNA polymerase (NEB). Similarly, overlap extension PCR was performed to amplify plant expression vector pB_35S/mEGFP (Addgene #135320) with ends that matched the ends of the Cas13 product so RfxCas13d expression would be under the control of 35S Cauliflower mosaic virus promoter. The PCR products were treated with DpnI (NEB), assembled together in a HiFi DNA assembly reaction (NEB), transformed into NEB10b cells (NEB), and grown overnight on antibiotic selection to create plasmid pB_35S/RfxCas13. Successful clones were identified and confirmed by sequencing followed by transformation into electro-competent *A. tumefaciens* strain GV3101 (pMP90).

#### Construction of crRNA expression vector

Single stranded oligonucleotides corresponding to “monovalent”, non-targeting (NT), and “polyvalent” gRNAs were purchased from Integrated DNA Technologies (Coralville, IA), phosphorylated, annealed, and ligated into binary vector SPDK3876 (Addgene #149275) that had been digested with restriction enzymes XbaI and XhoI (NEB) to be expressed under the pea early browning virus promoter (pEBV). The binary vector containing the right constructs were identified, sequenced and finally transformed into *A. tumefaciens* strain GV3101. Multiplexed expression of two crRNAs was achieved by ligating (annealed, phosphorylated) oligos for two individual crRNAs (hairpin + spacer) together with an internal 4 nt “sticky-end” and into SPDK3876 so both crRNAs would be expressed on a single transcript.

#### Agro-infiltration of *Nicotiana benthamiana* (tobacco) leaves

In addition to pB_35S/RfxCas13 and the SPDK3876’s harboring gRNA sequences (TRV RNA2), PLY192 (TRV RNA1) (Addgene #148968) and RNA viruses TRBO-GFP (Addgene # 800083) were individually electroporated into *A. tumefaciens* strain GV3101. Single colonies were grown overnight at 28 degrees in LB media (10 g/L tryptone, 5 g/L yeast extract, 10 g/L NaCl; pH 7). The overnight cultures were then centrifuged and re-suspended in infiltration media (10 mM MOPS buffer pH 5.7, 10 mM MgCl2, and 200 μM acetosyringone) and incubated to 3–4 hours at 28 degrees. The above cultures were mixed to a final OD600 of 0.5 for CasRX-NLS-GFP-pB35, 0.1 for PLY192 (TRV RNA1), 0.1 for RNA2- crRNAs and 0.005 for TRBO-GFP and injected into healthy leaves of five to six-week-old *N. benthamiana* plants grown under long-day conditions (16 h light, 8 h dark at 24°C). A total of four leaves for each gRNA were infiltrated. Three days after transfection, leaves were cut out and photographed under a handheld UV light in the dark, and stored at −80°C before subsequent analysis.

#### Quantitative RT-PCR

Total RNA was extracted from infiltrated leaves using RNeasy Plant Mini Kit (Qiagen) and the yield was quantified using a nanodrop. A total of 1 μg RNA from control (NT gRNAs) and experimental samples were used for DNase I treatment (Ambion, AM2222) followed by reverse transcription using a poly-dT primer and the Superscript III First Strand cDNA Synthesis System for RT–PCR (Invitrogen). Quantitative PCR was performed on Quant studio 3 Real-Time PCR System from Applied Biosystems using iTaq PowerUP^TM^ SYBR Green pre-formulated 2x master mix (Applied Biosystems). Relative expression levels based on fold changes were calculated using the ddCT method. Cycle 3 GFP mRNA expression levels from the TRBO-GFP replicon were normalized against transcripts of the tobacco PP2A, then to compare gRNAs across experiments, mRNA expression levels within a batch of plants are normalized relative to the mean GFP levels in experiments with non-viral targeting controls (dCas13d with no gRNA in Figure 2D and Cas13d with gNT in Figure 2E) performed at the same time as the other experimental conditions. The samples were performed in three biological replicates.

#### Cas13 collateral activity assays

Initial screens were performed using synthetic dsDNA (∼300 bp) containing a T7 promoter located upstream of a specific target sequence derived from either SARS-CoV-2 (Figures 3C and S7) or human *CD46* transcript sequences (Figure 3B) in two steps as follows: 1 μL *Leptotrichia wadei* Cas13a (LwaCas13a) enzyme (106 ng; Molecular Cloning Laboratories, South San Francisco, CA, USA) was preincubated with each pre-synthesized gRNA (CD46 targets) [0.25 μM; Integrated DNA Technologies, Coralville, Iowa, US (IDT)] or *in vitro* transcribed gRNA (SARS-CoV-2 targets) [0.25 μM; NEB] in a total volume of 5 μL for 10 min at room temperature, followed by the addition of 16 μL of synthetic dsDNA template (Twist Biosciences, South San Francisco, CA, USA) at varying concentrations (4.0 × 10^5^ cp/μL, 4.0 × 10^7^ cp/μL, or 4.0 × 10^9^ cp/μL at final concentration for SARS-CoV-2 targets and 1.0 × 10^9^ cp/ul for CD46 targets). A master mix containing 0.5 μL of T7 RNA polymerase [New England Biolabs, Ipswich, MA, USA (NEB)], 1 μL of 25 mM rNTPS (at equal ratios of rATP, rUTP, rGTP, rCTP; NEB), 0.23 μL 1M MgCl2 (Invitrogen Thermo Fisher, CA, USA), 0.5 ul HEPES (Invitrogen Thermo Fisher, CA, USA), 0.63 μL of RNAseH inhibitor (NEB), 1.56 μL RNAse Alert Reporter (IDT), and 0.58 ul of nuclease-free water (Invitrogen) were assembled on ice and 4 μL added to the mixture containing the DNA template and preincubated Cas13 RNP. 25 μL of each preassembled reaction was added to a 384-well plate (Black/Clear Bottom) and loaded into a preheated fluorescence microplate reader (Promega GloMax Explorer) at 37°C. Data readouts were collected every 5 min for 1hrat an excitation peak at 480 nm and an emission peak at 520 nm. List of all (p)gRNAs screened is found in Table S10: RfxCas13d pgRNAs for SARS-CoV-2 used in Collateral Activity assays and the results found in Table S11: Results of SARS-CoV-2 collateral activity assays.

Specificity of Cas13 collateral activity was evaluated using dsDNA fragments that were not complementary to the gRNAs being tested to confirm that activation of collateral activity as well as human universal RNA (10 tissues) (Invitrogen Thermo Fisher, CA, USA), and total human lung RNA (Invitrogen Thermo Fisher, CA, USA), was also used at 1 and 3 ug, respectively per reaction.

#### SHERLOCK-type viral detection reactions

Heat-inactivated SARS-CoV-2 RNA from respiratory specimens, deposited by the Centers for Disease Control and Prevention, was obtained through BEI Resources, NIAID, NIH: Genomic RNA from SARS-Related Coronavirus 2, Isolate USA-WA1/2020, NR-52285 (American Type Culture Collection (ATCC) VR-1986HK). In a SHERLOCK-type reaction, 1 μL of heat-denatured SARS-CoV-2 (350,000 copies total) was reverse transcribed using the High Capacity cDNA Reverse Transcription Kit (Thermo Fisher Scientific) with 3.4 μL of primer (0.5 μM) in a final volume of 16 μL and PCR-amplified by the addition of 2 μL of reverse and forward target primers (2 μM) and 20 μL of 2 x OneTaq Master Mix (NEB) in a final volume of 40 μL under standard thermocycler conditions (2 min at 95°C, followed by 35 cycles of 30 s at 95°C, 30 s at 49°C, and 30 s at 68°C, followed by a final extension of 5 min at 72°C). Reactions were diluted to 4, 40, or 400 cp/uL of the starting viral material (Myhrvold et al., 2018) for each target and Cas13 collateral activity assays were performed as described above. Collateral activity assays using Cas13 proteins with no gRNA were also evaluated to provide equivalent background signals.

#### Generation of Cas9 target sequences

Cas9 DNA targets in Figure 3 were generated by either (1) using plasmids containing targets listed in Table S12: Cas9 gRNAs, pgRNAs, and targets for *ex vivo* validation of the pgRNA design protocol, or PCR amplification of targets in plasmids using primers listed in Table S13: Oligos, DNA , and Genomic sequences used in this study.

Plasmids were purified using Monarch Plasmid Miniprep Kit following standard protocols (NEB, New England Biolabs, Ipswich, MA). PCR amplification was carried out using 4.5 ng of plasmid DNA, downstream and upstream PCR primers (IDT, Integrated DNA Technologies, Coralville, Iowa, USA) at a final concentration of 0.2uM, and Taq 2x Master Mix (NEB, New England Biolabs, Ipswich, MA, USA) following standard thermocycling protocols. Amplified PCR targets were purified using a Monarch PCR and DNA cleanup kit (NEB) following standard protocols. DNA oligonucleotides were hybridized to form duplex DNA targets by using equal molar concentration of oligos (Integrated DNA Technologies, Coralville, IA) to a final concentration of 10uM in nuclease-free IDT Duplex buffer. Reactions were heated to 95°C for 2 min and allowed to cool to room temperature prior to the reaction assembly.

#### *In vitro* transcription of Cas9 gRNAs

Single guide RNA (sgRNA) was synthesized by using the EnGen sgRNA synthesis Kit (New England Biolabs, Ipswich, MA, USA) following standard protocols. DNA oligos (IDT) were designed to contain a T7 promoter sequence upstream of the target sequences with an initiating 5′- d(G), as well as overlapping tracrRNA DNA sequence at the 3′ end of the target. The sgRNA was purified using Monarch RNA Cleanup Kit (NEB) and quantitated using standard protocols.

#### Duplex gRNA generation

Duplex CRISPR gRNAs (cRNA:tracrRNA) was generated by hybridizing synthetic RNA oligos listed in Table S12: Cas9 gRNAs, pgRNAs, and targets for *ex vivo* validation of the pgRNA design protocol to a universal synthetic tracer RNA oligo (IDT). To hybridize oligos, equal molar concentration of oligos were combined in IDT duplex buffer to a final concentration of 10uM. Reactions were heated to 95°C for 2 min and allowed to cool to room temperature prior to the reaction assembly.

#### Cas9 cleavage reactions

Cas9 Nuclease from *S. pyogenes* (NEB) was diluted in 1x NEB Buffer 3.1. prior to the reaction assembly. Cas9 cleavage activity was performed using either PCR-amplified targets, whole plasmid, or hybridized DNA oligos containing desired targets using standard methods. Briefly, Cas9 was preincubated with either a sgRNA or duplex gRNA (crNA:tracRNA) for 5 min at equal molar concentrations in 1x NEB Buffer 3.1 (NEB) in a volume total of 10 ul. Reactions were incubated for 5–10 min at room temperature. Target DNA was then added to the reactions, NEB Buffer 3.1 was added back to a final concentration of 1x, and nuclease-free water was added bringing the final volume to 20 ul. The final reaction contained 100 nM Cas9-CRISPR complex and 10 nM of target DNA. Similar reactions without the addition of gRNAs to Cas9 were used as a control for uncut DNA. Reactions were incubated at 37°C for 1 h, followed by the addition of 1 unit of Proteinase K and further incubation at 56°C for 15 min. Reactions were stopped by the addition of one volume of purple Gel Loading dye (NEB).

Fragments were separated and analyzed using a 1.5% Agarose gel in 1xTAE and 1X SYBR Green 1 Nucleic Acid Gel Stain (Thermo Fisher Scientific; Waltham, MA), and fluorescence was photographed and measured (AmershamTM Imager 600; GE Life Sciences, Piscataway, NJ, USA). Cas9 cleavage efficiency was calculated from fluorescence intensity at each band.

#### Estimation of SARS-CoV-2 target sequence conservation

All complete SARS-CoV-2 genomic sequences available from the NCBI Virus database were downloaded on November 23, 2020 (29,123 sequences). For each of the 205 target pairs possessing biophysically feasible pgRNA candidates, we aligned (no gaps) each target sequence to each genome to determine the closest matching sequence. Alignments containing ambiguous nucleotide calls were not included. Sequence variants were grouped together, with a minimum prevalence of 0.1%, with the fraction of hits by the most prevalent group being considered the sequence conservation reported.

### Quantification and statistical analysis

Data was quantified using the methods described above, and the statistical analysis was performed using Microsoft Excel and MATLAB (MathWorks, Inc.). The numbers of samples (N) are plotted explicitly in each graph and/or recorded explicitly in the supplementary table where the analysis is performed. Hypothesis testing regarding differences in the mean quantities of viral RNA were performed in MATLAB using two-sided t-tests.

## Data Availability

• *Data:* all data generated or analyzed during this study are included in this article and its supplementary information files. Any additional information required to re-analyze the data reported in this article is available from the lead contact on request. • *Code:* Our computational tools for designing pgRNAs for Cas9 and Cas13 are publicly available at https://www.github.com/ejosephslab/pgrna (https://doi.org/10.5281/zenodo.7126522) as of the date of publication. DOIs are listed in the key resources table. • *All other requests*: All other requests should be directed to and will be furnished by the lead contact, Eric Josephs (eric.josephs@uncg.edu).

## Supplemental information

Document S1. Figures S1–S10

Table S1. Statistics of Homeologous Cas13 Target Pair (>16/23 or 70% sequence identity) Prevalence in RNA viral genomes, related to Figure 1

Table S2. Homeologous Cas13 Target Pairs (>16/23 or 70% sequence identity) of Human-hosted RNA viruses, related to Figure 1

Table S3. Statistics of pgRNA Candidate Prevalence in human-hosted (+) ssRNA viral genomes and viral RNA transcripts, related to Figure 1

Table S4. pgRNA Candidate Prevalence in human-hosted (+) ssRNA viral genomes and viral RNA transcripts, related to Figure 1

Table S5. RfxCas13d pgRNAs for TRBO-GFP and Associated Properties of the pgRNAs, related to Figure 2

Table S6. RfxCas13d gRNAs and pgRNAs used in N. bethamiana, related to Figure 2

Table S7. RT-qPCR of GFP mRNA in N. bethamiana after transient expression of TRBO-GFP; RfxCas13d; and gRNAs, pgRNAs, or non-targeting (NT) gRNAs, related to Figure 2

Table S8. RfxCas13d pgRNA Target Pairs and Sequence Conservation for SARS-CoV-2, related to Figure 3

Table S9. RfxCas13d pgRNAs for SARS-CoV-2 and Associated Properties of the pgRNAs, related to Figure 3

Table S10. RfxCas13d pgRNAs for SARS-CoV-2 used in Collateral Activity assays, related to STAR Methods

Table S11. Results of SARS-CoV-2 collateral activity assays, related to STAR Methods

Table S12. Cas9 gRNAs, pgRNAs, and targets for ex vivo validation of the pgRNA design protocol, related to Figure 4

Table S13. Oligos, DNA , and Genomic sequences used in this study, related to STAR Methods
