# Supplementary material for: Polyvalent guide RNAs for CRISPR antivirals
Source: iScience. 2022 Oct 13;25(11):105333. doi: 10.1016/j.isci.2022.105333 (PMC9618770; doi:10.1016/j.isci.2022.105333)
Supplement: Document S1. Figures S1–S10 [file mmc1.pdf]

iScience, Volume *25*

Supplemental information

Polyvalent guide RNAs for CRISPR antivirals

Rammyani Bagchi, Rachel Tinker-Kulberg, Mohammad Salehin, Tinku Supakar, Sydney Chamberlain, Ayalew Ligaba-Osena, and Eric A. Josephs

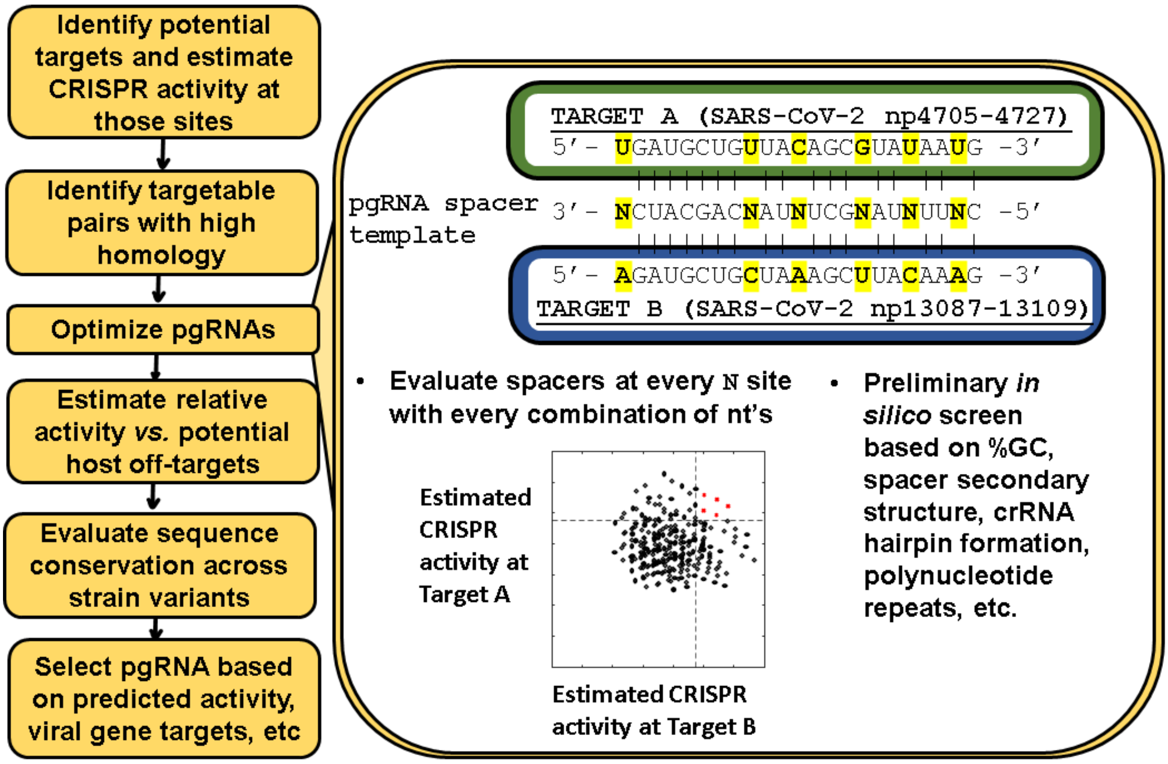

Figure S1. Workflow for the computational design of polyvalent guide RNAs (pgRNAs). Related to Figure 1C.

Briefly, after all possible targets in the viral genome are evaluated for CRISPR predicted activities, using tools such as sgRNA Designer for Cas9[1, 2] or cas13design for Cas13d,[3] pairs of targetable sequences in the top quartile of predicted on-target activity with large fractions of identical sequence (*e.g.*, ≥70%) are identified. A pgRNA spacer template is generated (right). For pairs with n sites where the sequence differs, 4n candidate pgRNA spacers are generated with every possible combination of nucleotides at those n sites, which are then evaluated for sufficient predicated relative activities at both target pairs using a Cutting Frequency Determination (CFD) score. They are then screened in silico for acceptable biophysical properties known to affect CRISPR activity (secondary structure, GC%, etc.). Those pgRNA candidates with acceptably high relative activity across all clinical strain variants and acceptably low predicted activity at potential off-target sites with the human genome/transcriptome can then be further screened for additional criteria (targeting specific genes or regions of interest (ROIs), for examples) and evaluated using additional gRNA design tools or validated experimentally. Implementation of this algorithm in Python is available at: https://github.com/ejosephslab/pgrna.

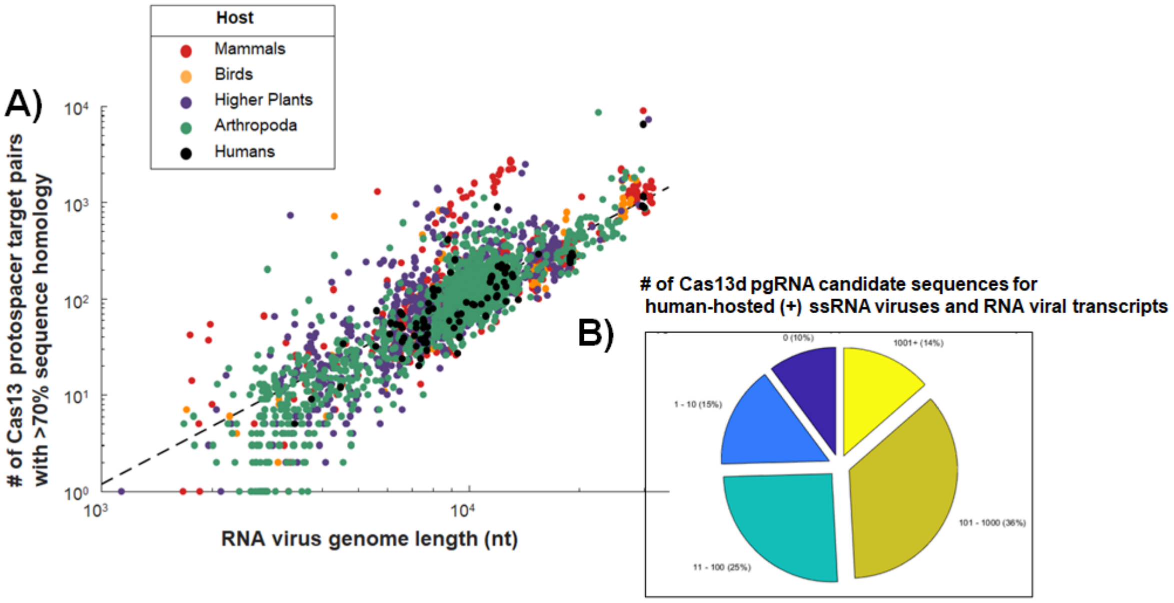

Figure S2. Statistics of Homeologous Cas13 Target Pairs (>16/23 or 70% sequence identity) Prevalence in RNA viral genomes. Related to Figure 1D.

(A) Pairs of targetable sites for Cas13 (23 nt), which share at least 70% homology, are abundant across the genomes of RNA viruses. The number of target pairs (P) vs. genome length (L) is fit well (R^2 = 0.7288) by: $\log_{10}(P) = 2.202 * \log_{10}(L) - 6.76$, or equivalently $P = 1.74e\text{-}7 * L^{2.202}$. All complete, RefSeq-quality genomes of RNA viruses, excluding proviruses, available by December 27, 2020 were downloaded from the NCBI Virus database with hosts: arthropoda (1074 viral species), aves (111), mammal (496), higher plant / embrophyta (691), and human (89). Genomes composed of multiple segments or CDS from the same viral isolate were considered together. (B) 53 of 59 human-associated (+) ssRNA viruses or RNA viral transcripts have at least 1 candidate pgRNA sequence (with 50% many more having >100 candidates sequences). Candidate pgRNAs sequences defined as having predicted activity at multiple viral sites to be in the top quartile of all anti-viral gRNAs; having no BLAST hits to human transcriptome (no complementary targets with >15 out of 23 nt complementarity); and having no biophysical characteristics like strong secondary structure or mononucleotide repeats that would inhibit activity or expression. See Supplementary tables S3 and S4, while noting that these predicted pgRNAs have not been validated experimentally for on- or off-target activity (unless explicitly noted in manuscript).

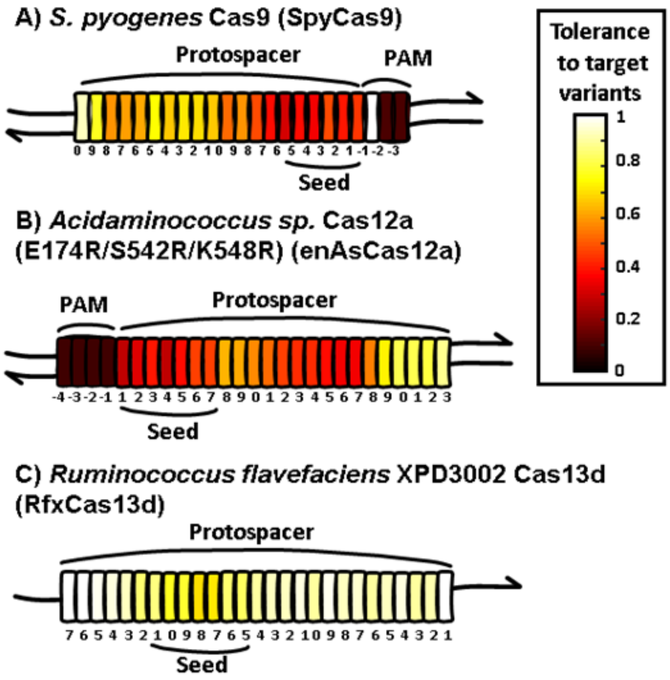

Figure S3. Position-dependent effects of sequence variants in the targeted region on the activity of different CRISPR effectors. Related to Figure 1C. (A) type II CRISPR effector Cas9 from *S. pyogenes* [4, 5], which targets dsDNA; (B) an engineered variant of type V CRISPR effector Cas12a from *Acidaminococcus sp*. BV3L6, enAsCas12a [6, 7], which targets dsDNA; and (C) type VI CRISPR effector Cas13d from *Ruminococcus flavefaciens* XPD3002 [3, 8], which targets ssRNA. Targeted nucleotides are coloured by the tolerance, or the average change in activity if there is a sequence variation at that site, of the CRISPR effectors for each position within or near the targeted (protospacer) sites. Tolerance of 1 implies that the CRISPR effector exhibits no change in activity regardless of the nucleotide identity at that site, and a tolerance of 0 implies that CRISPR activity is completely abolished if the nucleotide sequence is changed from the expected nucleotide at that site. The “seed” region is defined as a region of high sensitivity (low tolerance) to sequence variations at those sites; Cas9 and Cas12a are also highly sensitive to sequence variations at the protospacer adjacent motif (PAM) that are recognized by the enzyme itself rather than the gRNA. For the design of polyvalent gRNAs, we seek to maximize activity of a single gRNA at multiple viral sites by exploiting well-tolerated mismatch- and position- specific mispairings of the CRISPR effectors to minimize potential reductions of activity at these different sites.

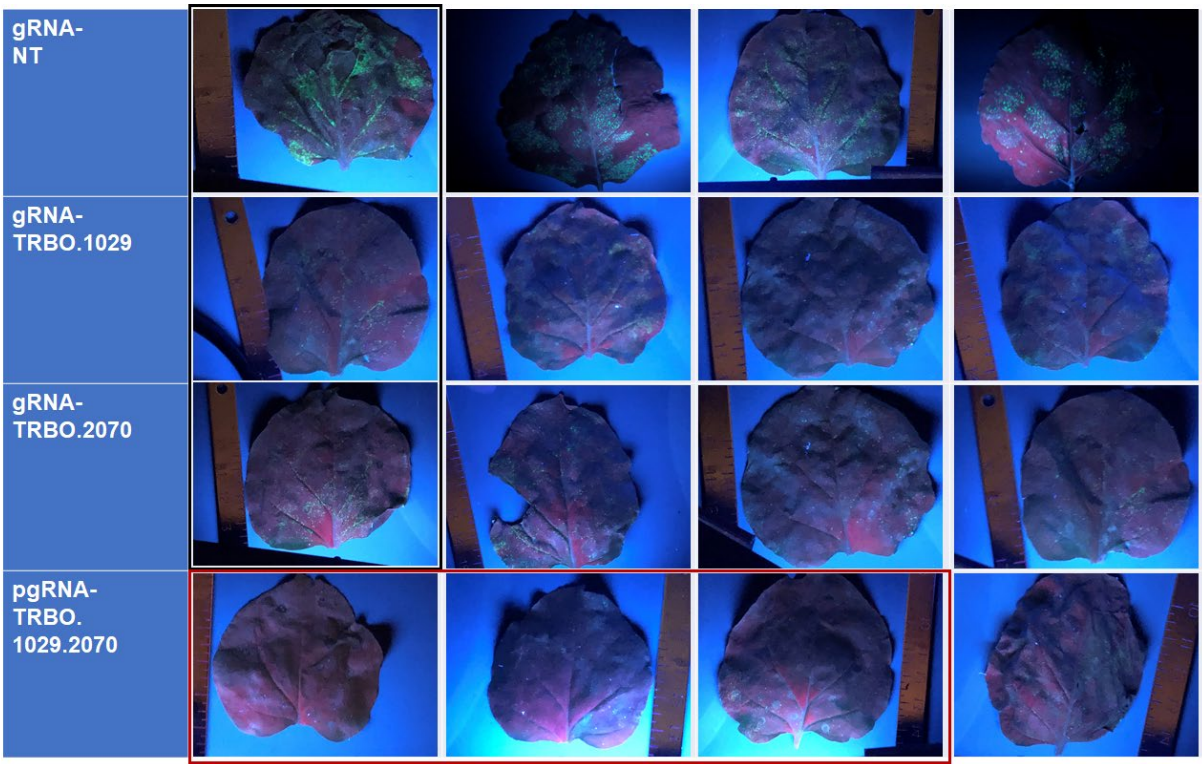

Figure S4. Additional photographs of leaves after induction of TRBO-GFP proliferation and transient expression of RfxCas13d with different gRNAs or pgRNAs. Related to Figure 2.

Images boxed in black and red are the same images as shown in Figure 2C in their respective colors.

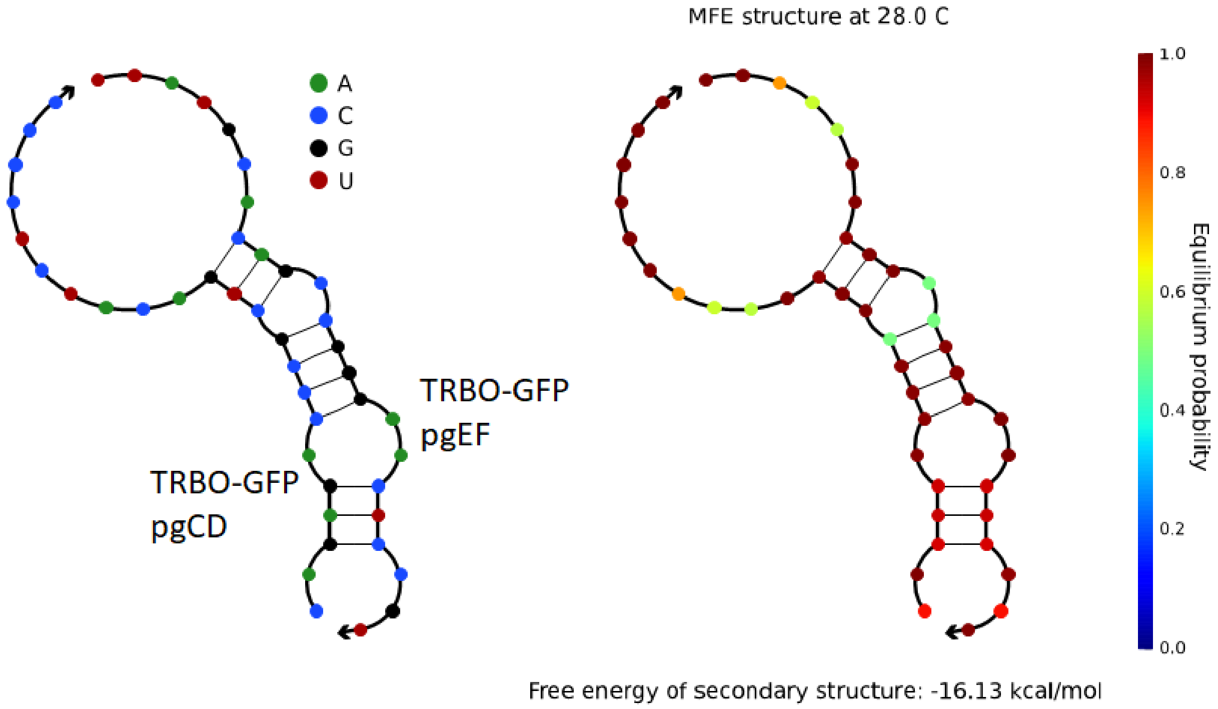

Figure S5. Predicted interaction between TRBO-GFP pgCD and pgED. Related to Figure 2E.

(Left) Sequence identity and (right) mean free-energy (MFE) probabilities of predicted base-pairing interactions between the TRBO-GFP pgRNAs “pgCD” and “pgEF” predicted using the NUPACK suite.[9] No other stable base-pairing interactions between multiplexed pgRNAs were predicted.

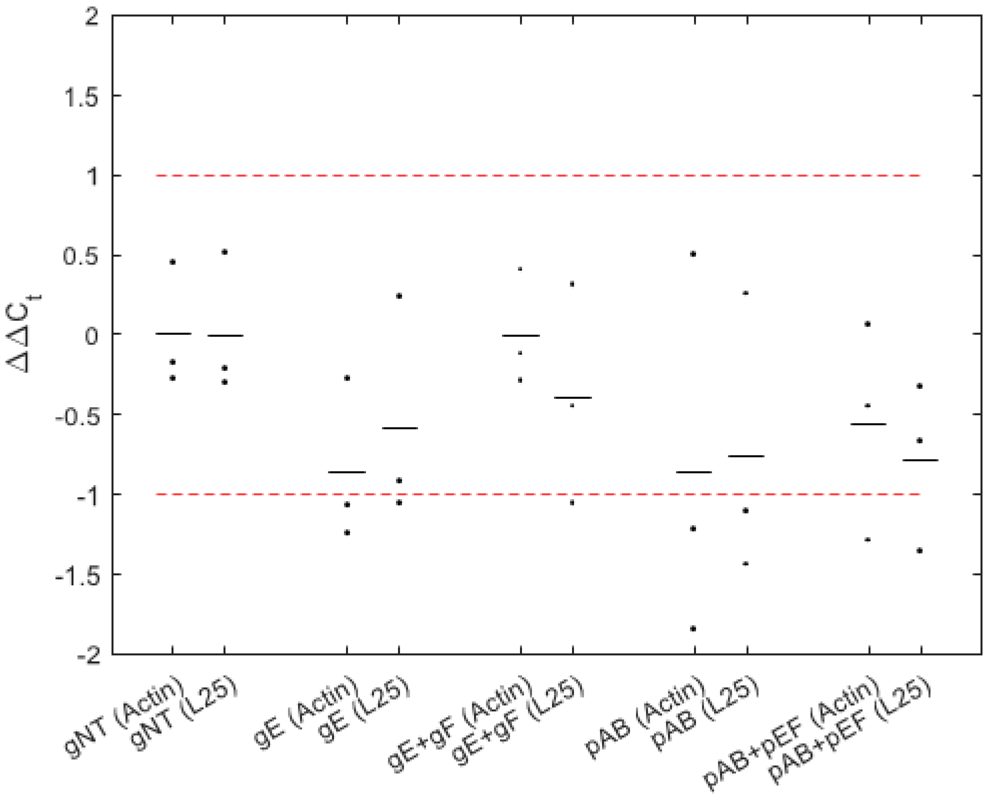

Figure S6. RT-qPCR of two other householder genes (*ACTIN* and *L25*) reveals no evidence of "off-target" activity by Cas13 with different gRNAs targeted to the TRBO-GFP. Related to Figures 2C-E.

Results are relative to levels of householder *PP2A* mRNA in *N. benthamiana.* pgRNAs were screened to ensure they all have at least 8 mismatches out of 23 nt spacers with any complements of the *N. benthamiana* transcriptome.

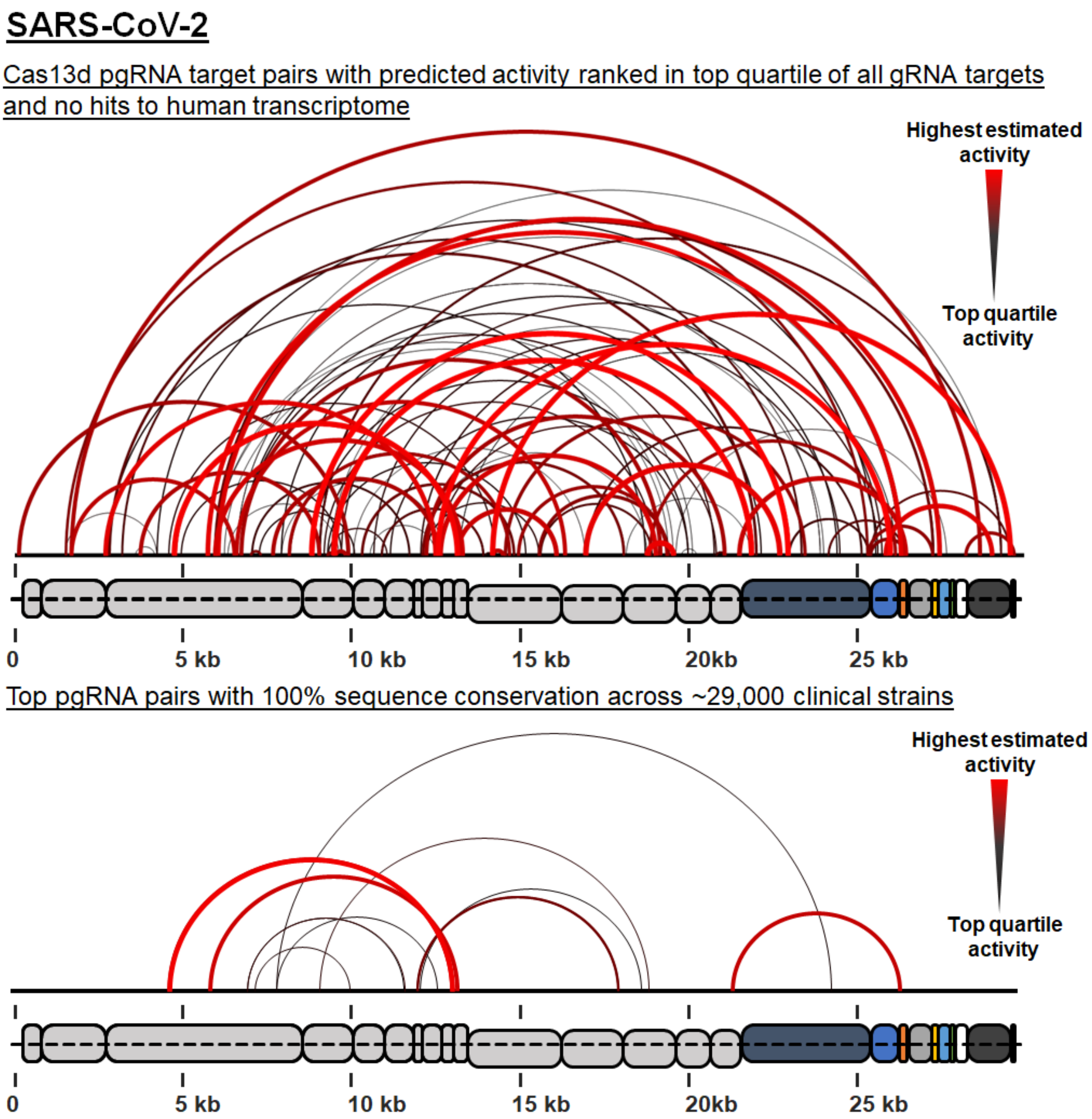

Figure S7. Target pairs in the SARS-CoV-2 genome for pgRNAs. Related to Figure 3.

144 pairs of which predicted to have activity ranked in the top quartile of all anti-SARS-CoV-2 gRNAs at both sites and no predicted reactivity with the human transcriptome (top), 15 of which (bottom) target sites both with 100% sequence conservation across ~29,000 sequenced clinical variants.

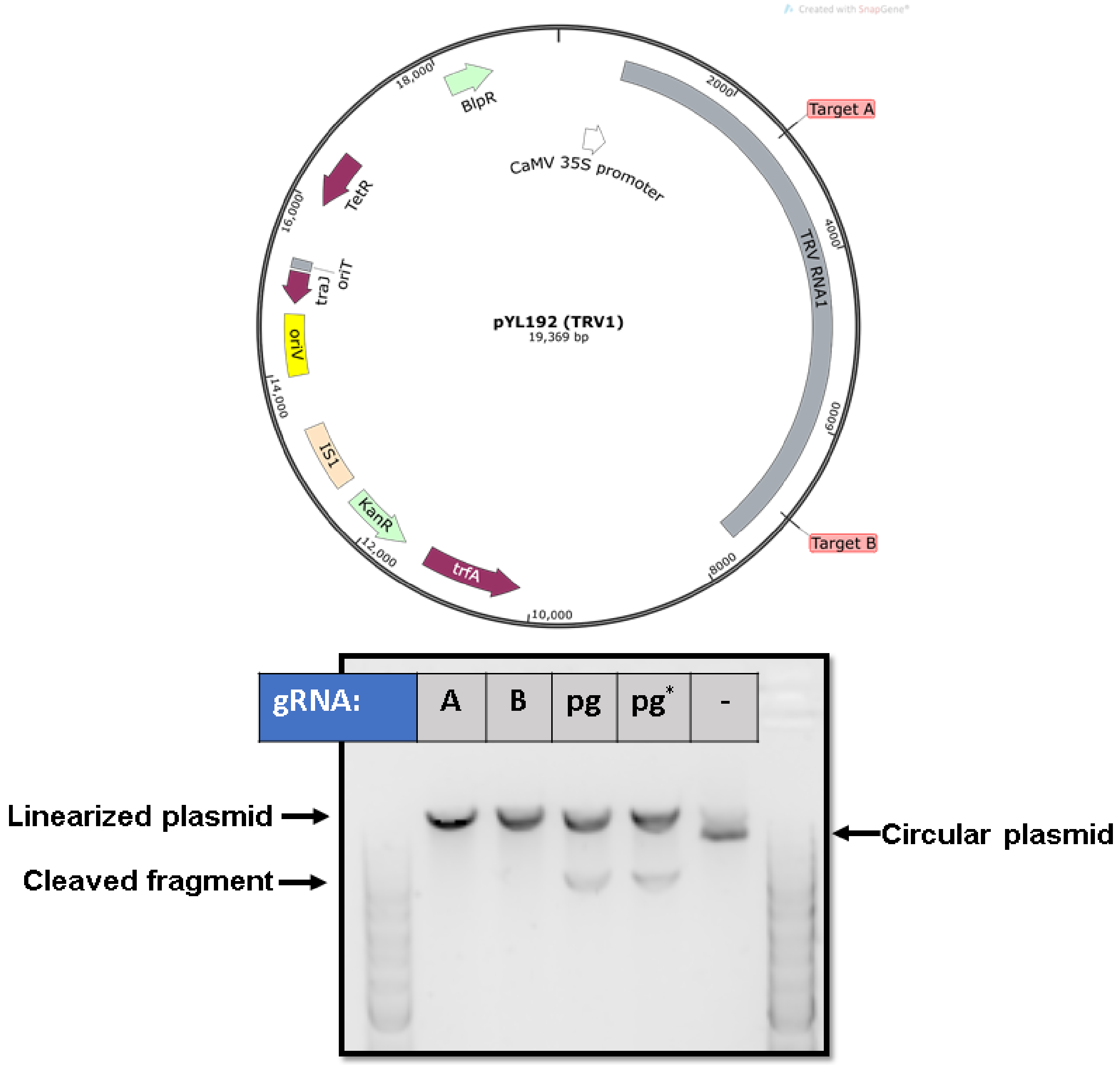

Figure S8. Simultaneous cleavage of two targets on the same plasmid by a Cas9-pgRNA RNP *in vitro*. Related to Figure 4.

(Above) Plasmid map with targets (see Figure 4B) in red are located ~4kbp apart. (Below) Agarose gel electrophoresis after incubation of the plasmid and Cas9 with a “monovalent” gRNA specific to target A (A), a “monovalent” gRNA specific to target (B), or a “polyvalent” gRNA (pgRNA) optimized for activity at both targets. The sample marked with an * was incubated with and additional 1% polyethylene glycol during the cleavage reaction.

Target	T. 7	T. 7	T. 8	T. 8	uncut	T. P.	T. 7	T. 8	T. 5	T. 6	T. 3	T. 4	T. 1	T. 2
gRNA	gRNA7	gRNA8	gRNA8	gRNA7	-	pgRNA	pgRNA	pgRNA	pgRNA	pgRNA	pgRNA	pgRNA	pgRNA	pgRNA
% Cleaved	75.1%	0%	74.%5	0%	n/a	78.2%	0%	88.2%	32.4%	79.7%	70.5%	72.7%	71.3%	63.5%

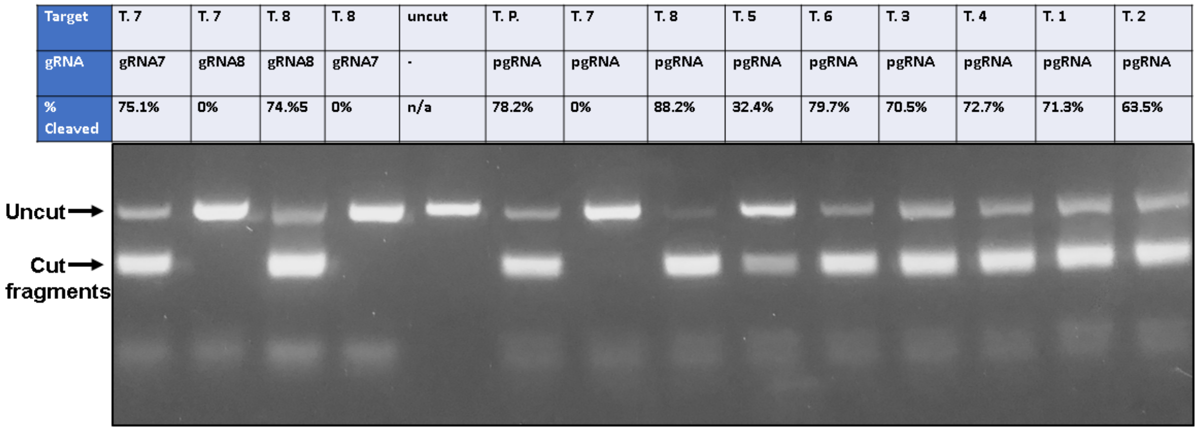

Target	HIV 4794	HIV 9078	HPV16 2716	HPV16 7434	HTLV1 4382	HTLV1 7503	HAvC 9382	HAvC 11125	HPV16 4525	HPV18 934	HAvC 731	HAvC 25247	HIV 4794
gRNA	pgRNA-HIV. 4794s. 9078s	pgRNA-HIV. 4794s. 9078s	pgRNA-HPV16. 2716s. 7434a	pgRNA-HPV16. 2716s. 7434a	pgRNA-HTLV1. 4382a. 7503a	pgRNA-HTLV1. 4382a. 7503a	pgRNA-HAvC. 9382a. 11125a	pgRNA-HAvC. 9382a. 11125a	pgRNA-HPV16. 4525a. HPV18. 934s	pgRNA-HPV16. 4525a. HPV18. 934s	pgRNA-HAvC. 731s. 25247s	pgRNA-HAvC. 731s. 25247s	-
% Cleaved	26.6%	46.2%	43.9%	61.3%	44.9%	0%	0%	47.1%	62.1%	0%	53.4%	64.5%	n/a

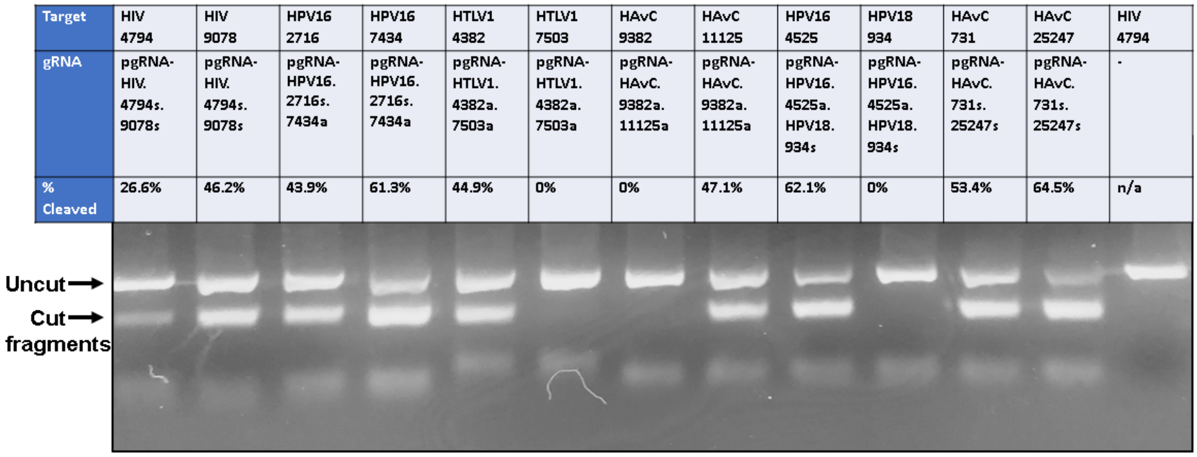

Figure S9. Agarose gel electrophoresis of Cas9-pgRNA cleavage products of synthetic targets with increasing divergence; related to Figure 4D.

Sequences can be found in Supplementary Table S12.

Target	A	A	A	B	B	B	A	A	A	B	B	B	A	A	A	B	B	B
gRNA	gA	gA	gA	gA	gA	gA	gB	gB	gB	gB	gB	gB	pg	pg	pg	pg	pg	pg
gRNA structure	sg	dupl.	dupl.	sg	dupl.	dupl.	sg	dupl.	dupl.	sg	dupl.	dupl.	sg	dupl.	dupl.	sg	dupl.	dupl.
5’ G	+	-	+	+	-	+	+	-	+	+	-	+	+	-	+	+	-	+
% Cleaved	87	74	85	0	0	0	0	0	0	86	72	81	59	82	81	34	61	35

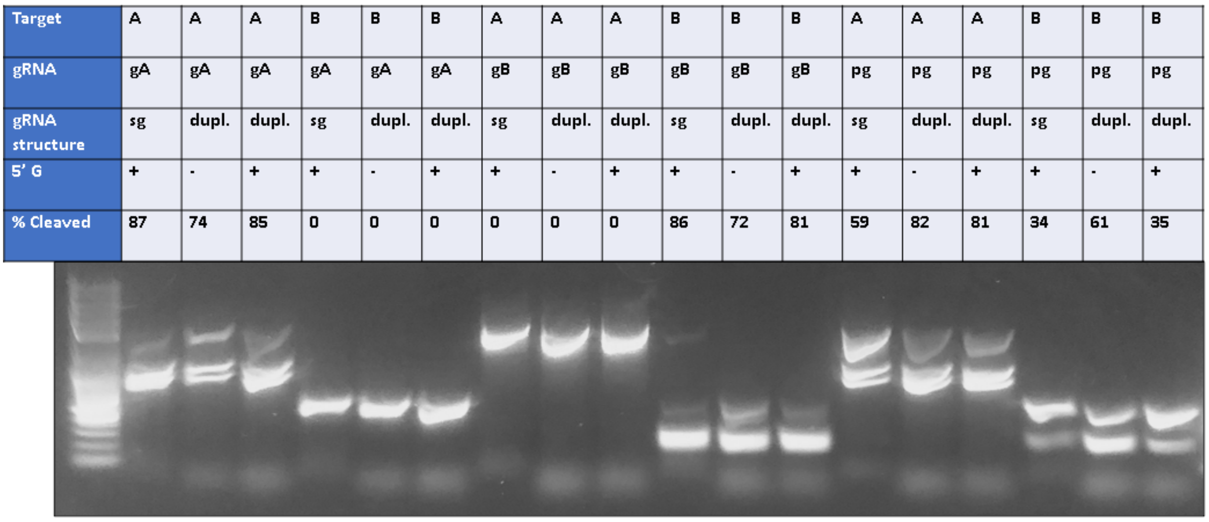

Figure S10. Agarose gel electrophoresis of Cas9-gRNA cleavage products of targets in Figure 4B under different *in vitro* conditions typically optimized for gene editing. Related to Figure 4B.

Those gRNAs marked with 5’- have 21 nt long spacers with an unpaired 5’- G. Those with ‘sg’ gRNA structures were transcribed *in vitro* as ‘single guides RNAs’ (sgRNA) that fuse the crRNA and tracrRNA in continuous RNA molecule, while for ‘dupl.’ (duplex) the crRNA was synthetized (IDT; Coralville, IA) and hybridized with a tracrRNA (IDT) prior to incubation with the Cas9. Sequences can be found in Supplementary Table S12.
